# Decoding Proton‐Coupled Electron Transfer Mechanism of Nicotine for Multi‐Scenario Portable Electrochemical Sensing

**DOI:** 10.1002/advs.202521198

**Published:** 2025-12-22

**Authors:** Yi Peng, Qinyi Cao, Qianyu Shen, Yuhang Zhang, Shiyu Hu, Hongdou Yi, Qian Liu, Lihui Ou, Qiang Li, Zhaohong Su

**Affiliations:** ^1^ College of Chemistry and Materials Science College of Agronomy Hunan Agricultural University Rapeseed Variety Creation Center Team，Yuelushan Laboratory Changsha China; ^2^ College of Chemistry and Materials Engineering Hunan University of Arts and Science Changde China; ^3^ State Key Laboratory of Environmental Chemistry and Ecotoxicology Research Center for Eco‐Environmental Sciences Chinese Academy of Sciences Beijing China

**Keywords:** nicotine, electrooxidation mechanism, portable sensor, proton‐coupled electron transfer, density functional theory

## Abstract

A key challenge in nicotine electroanalysis is the unresolved complexity of its interface process, which directly determines sensor metrics. We have developed a screen‐printed analysis strip to elucidate potential proton‐coupled electron transfer processes, while achieving multi‐scenario nicotine detection (tobacco leaves, dry tobacco leaves, cigarettes, and smokers' saliva). The electrochemical characterization of elucidated the pH‐dependent (pH 5–9) 2H^+^/2e^−^ coupling transfer mechanism of nicotine. Density functional theory calculations revealed molecular‐level insights: Electrostatic potential and Fukui function revealed the differences in electronic structure characteristics of nicotine molecule in different protonated states and their regulatory in the electrooxidation process; Quantitative characterization of spontaneous protonation thermodynamics (Δ*G*_1_ = −29.36 kcal/mol, Δ*G*_2_ = −17.68 kcal/mol) and interfacial electron transfer (0.0219 e^−^) provided theoretical foundations for reaction kinetics; Transition state analysis uncovered a hydroxyl‐dominated multilevel synergistic electrooxidation mechanism. This integrated platform not only decodes the complex interface mechanism of nicotine but also establishes a universal research paradigm for proton‐coupled systems, demonstrating successful applications from industrial quality control to biomedical monitoring.

## Graphical abstract

A key challenge in nicotine electroanalysis is the unresolved complexity of its interface process, which directly determines sensor metrics. Herein, the proton‐coupled electron transfer mechanism of nicotine is decoded for multi‐scenario portable electrochemical sensing. This integrated platform establishes a universal research paradigm for proton‐coupled systems, demonstrating successful applications from industrial quality control to biomedical monitoring.

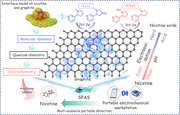

## 1 Introduction

Nicotine (NIC) is an alkaloid found in Solanaceae plants. As the main chemical component of tobacco, NIC is a toxic and highly addictive substance [1]. It can produce a series of physiological and psychological effects by stimulating the central nervous system, including refreshing, improving attention, enhancing mood, and inducing lung cancer and cardiovascular‐related diseases [2]. Long‐term use and dependence on NIC can seriously affect health [3]. Therefore, it is urgent to develop a new NIC rapid detection technology based on the needs of informatization and modernization, so as to overcome the bottleneck of on‐site and real‐time monitoring technology in the field of industrial process monitoring and biomedical diagnosis, alleviate the increasing pressure to achieve precise control of NIC content, and provide tissue, cell or organelle specific information of tobacco in real time [4].

The continuous updating of new technologies is an inevitable trend in the development of NIC detection, such as field effect transistor [5], capillary electrophoresis [6], supercritical fluid chromatography [7], vacuum ultraviolet photoionization aerosol mass spectrometry [8], fluorescence [9], and gas chromatography‐tandem mass spectrometry [10], etc. Although the traditional method has the disadvantages of cumbersome operation, a long time, and uneconomical, its accuracy is still the key guarantee to support the new method. The contradiction and cooperation between the two are the current situation in the field. The electrochemical method is favored by researchers because of its fast response and high precision, and can realize the rapid conversion between electrical signals and chemical signals, which greatly increases the amount of information available in all space–time dimensions, such as smart agriculture and biomedicine [11, 12, 13]. At the same time, the screen‐printed analytical strip (SPAS) came into being. Because of its multi‐functionality, small size, low cost, portability, and other advantages, it has been reused in the combination of sensors and nanomaterials [14, 15]. Due to the unique nature of SPAS, the electrochemical laboratory experiment can be transferred to the field detection of various analytes [16]. Currently, there is a key technical problem: what kind of electrochemical behavior does NIC exhibit on SPAS? This is not only the focus of on‐site detection, but also the key to achieving efficient and accurate detection of NIC.

The complexity of the electrochemical oxidation reaction stems from its microscopic dynamic process occurring at the electrode–electrolyte interface. This process has highly transient characteristics and is difficult to achieve real‐time on‐site monitoring. This characteristic constitutes the core scientific challenge faced by electrochemical sensing technology. At the same time, it also faces many external factors. The oxidation process of NIC in the high potential region is easy to cause electrode passivation, [17] and its electrochemical window overlaps with bioactive substances, resulting in selective interference. The pH of the solution significantly affects the oxidation mechanism by regulating the proton‐coupled electron transfer (PCET) [18] pathway. On the other hand, the differences in the generation mechanisms of reactive oxygen species, such as hydroxyl radicals (·OH) and peroxides (OOH^−^) in different pH media [19], may lead to complex NIC oxidation reactions. Chaya et al. found that NIC is prone to monoprotonation and diprotonation under different pH conditions, and there is an acid–base balance of NIC [20]. H. B. Suffredini et al. verified the electrochemical oxidation process of NIC at the optimal pH 8 and the number of electron and proton transfer by combining theoretical calculation with electrochemical experiments [21]. This study lacks systematic verification of electrode kinetic evolution and oxidation intermediate stability in a wide pH range. Although the transfer of two protons and two electrons in NIC is widely accepted by researchers, the study by Elif Turker Acar [22] and Pedro H.S. Borges [23] proposed a single proton, single electron pathway. Their work crucially demonstrates that electrode materials are active participants in the oxidation process, indicating that the observed pathways are highly sensitive to the experimental interface. Although subsequent researchers have made progress in the modification of sensing materials, the mechanism of NIC electrooxidation is still unclear, especially the structure–activity relationship between the adsorption configuration of different protonated molecules at the electrode interface, and the charge transfer efficiency has not been elucidated [24, 25]. The intermediates that may be generated during the oxidation process [26] and their multilevel synergistic reaction pathways have not been effectively characterized. Therefore, it is necessary to systematically analyze the intrinsic relationship between the evolution of NIC molecular configuration and the volt–ampere response characteristics, revealing the matching mechanism between the orbital energy level of NIC molecules and the Fermi energy level of the sensing interface under protonation state regulation.

This work deeply deciphered the electrooxidation mechanism of NIC at the SPAS interface by coupling electrochemical analysis with quantum chemical calculations. Based on the electrochemical behavior of NIC, the reaction following the two‐electron two‐proton process was accurately quantified, and the key role of single/double protonation forms in the reaction was identified. By using density functional theory, the pH‐dependent reaction pathway and the interface electron transfer mechanism of NIC were elucidated at the molecular level, successfully extending the transition state theory to the complex oxidation process of NIC. And clarifying that the PCET mechanism belongs to the outer‐sphere PCET deepens the understanding of the reaction mechanism. This breakthrough at the mechanistic level directly guides the application boundaries of the two detection modes of the sensing system, significantly promoting the application of NIC detection methods in various industries and providing a key theoretical and practical basis for their cross‐disciplinary applications.

## 2 Results and Discussion

### 2.1 Characterization of SPAS Portable Sensor

There are strict requirements for the design distance of the sensing interface electrode, because different electrode spacing will lead to different current densities, resulting in different sensing performance, which also ensures the standardization and rigor of the experiment [27]. The dimensions and constituent materials of the SPAS are illustrated in Figure S1A. Based on virtual simulation technology, the working electrode (WE), reference electrode (RE), and counter electrode (CE) were modeled. The primary function of the RE is to complete the circuit and maintain the constant voltage generated by the CE and the WE [27]. The electrochemical analysis function is fulfilled by WE and CE. The morphology of WE and the parts between WE and CE were analyzed by SEM. It can be clearly observed that WE is composed of a graphite structure (oxidation‐reduction reactions occur on the electrode surface in this area), and there is only a smooth insulating layer between WE and CE (Figure S1B). Meanwhile, the addition of NIC does not affect the hydrophobicity of the SPAS interface (Figure S1C). This property is particularly advantageous for the detection of small molecules that are controlled by diffusion, especially for organic small molecules lacking hydrophilic functional groups. In addition, as shown in Figure S1D, XPS captured the elemental composition information of the graphite structure in the WE region, where elements C, O, and Si accounted for 76.05%, 18.48%, and 5.47%, respectively. Among them, the Si signal comes from the substrate material of the electrode. Moreover, the low signal intensities of Si2s and Si2p detected indicate that they only originate from the substrate background rather than the electrode active coating. Therefore, we only performed peak fitting on the dominant C1s and O1s orbitals in the spectrum that originate from the electrode active coating. In Figure S1E, four primary peaks at 284.78, 285.28, 286.78, and 289.28 eV correspond to C─C, C─O, C═O, and O─C═O, respectively. In Figure S1F, two main peaks at 532.28 and 533.38 eV correspond to C─O and C═O, respectively. SPAS was electrochemically characterized using a portable electrochemical workstation, with cyclic voltammetry (CV) and impedance spectroscopy (EIS) tests conducted in a 5.0 mm [Fe(CN)_6_]^3−/4−^ + 0.1 m KCl solution. As demonstrated in the CV results presented in Figure S1G, the potential difference between the two peaks of SPAS is 478 mV. At the oxidation peak, electrons are lost, while at the reduction peak, electrons are gained, completing an oxidation–reduction process. The symmetry of the oxidation–reduction peaks also indicates the reversibility of the electrochemical process. By integrating the curve area of the entire process, we can calculate that the charge passed through this process is Q = 6.22 × 10^−5^ C. The EIS data depicted in Figure S1H provide insights into the electrode interface characteristics and the Randles equivalent circuit model. In this model, the constant phase element (CPE) represents the capacitive behavior of the electrical double layer, while *R*_S_ denotes the resistance of the electrolyte solution. The impedance element *W* signifies the Warburg impedance, and *R*_ct_ corresponds to the charge transfer resistance, which is indicative of the electrochemical signal changes. Within the high‐frequency region of the semicircular Nyquist plot, the electron transfer is relatively minimal. The specific values obtained from the EIS experiment are as follows: the capacitance of the double layer (CPE) is 964.7 nF, the internal resistance of the solution (*R*_s_) is 72.82 Ω, and the charge transfer resistance (*R*_ct_), represented by the diameter of the high‐frequency semicircle in the Nyquist plot, is 4639 Ω. The heterogeneous electron transfer rate constant can be derived using Equation 1 [28].

(1) $$K^{0}=\frac{RT}{CF^{2}R_{ct}}$$

where *K* ^0^ is the heterogeneous electron transfer rate constant, *R* is the gas constant (8.314 J/(mol·K)), *T* is the Kelvin temperature (298.15 K), *F* is the Faraday constant (96485 C/mol), *C* is the [Fe(CN)_6_]^3−/4−^ concentration (5.0 mM), and *R_ct_* is the charge transfer resistance. The calculated *K* ^0^ of SPAS is 1.15×10^−7^ cm/s. To substantiate the reliability of the aforementioned CV test outcomes and to pave the way for the widespread market adoption of SPAS, it is imperative that SPAS demonstrate exceptional stability. Consequently, SPAS underwent 50 cycles of CV scanning in [Fe(CN)_6_]^3−/4−^ + 0.1 M KCl. The redox peak current values from these 50 cycles are meticulously analyzed (Figure S1I). The results show a high degree of consistency in the 50 cycles of CV, with RSD values of 1.19% and 2.09% for the oxidation and reduction peak current, respectively. The peak current maintains a relatively stable value, highlighting the excellent stability of SPAS [29].

The electrochemical behavior of SPAS was assessed using both a positive probe [Ru(NH_3_)_6_]^2+/3+^, [30] and a negative probe [Fe(CN)_6_]^3−/4−^, across a range of scanning rates from 40 to 180 mV/s. These measurements were conducted to determine the effective area of electrical activity, providing insights into the electrochemical properties of SPAS [31]. At the same time, it is proven that the positive and negative charges caused by protonation or deprotonation of the sensing object in different environments also have electrochemical behavior. The reversible redox peaks of the negative probe [Fe(CN)_6_]^3−/4−^ are depicted in Figure S1J, and a similar phenomenon is observed in the electrochemical tests of the positive probe [Ru(NH_3_)_6_]^2+/3+^ as shown in Figure S1K, with negligible differences in reversibility between the two probes. Additionally, it was discovered that the peak current (*I*_pa_) is directly proportional to the square root of the scanning rate (Figure S1L). This observation indicates that the Randles–Sevcik equation [32] (Equation 2) is being adhered to, further validating the electrochemical behavior under study [33]. It also shows that the mass transfer process of SPAS is controlled by diffusion.

(2) $$I_{p}=2.69\times 10^{5}n^{3/2}AD^{1/2}v^{1/2}C_{0}$$

where *n* is the number of transferred electrons, *A* is the electroactive surface area, *D* is related to the diffusion coefficient, *v* is the scanning rate, *C_0_* is the concentration of [Fe(CN)_6_]^3−/4−^ and [Ru(NH_3_)_6_]^2+/3+^. The electroactive area of SPAS for the negative probe is 0.060 cm^2^, and that for the positive probe is 0.124 cm^2^.

### 2.2 Electrochemical Behavior of NIC on SPAS Portable Sensor

**FIGURE 1:**
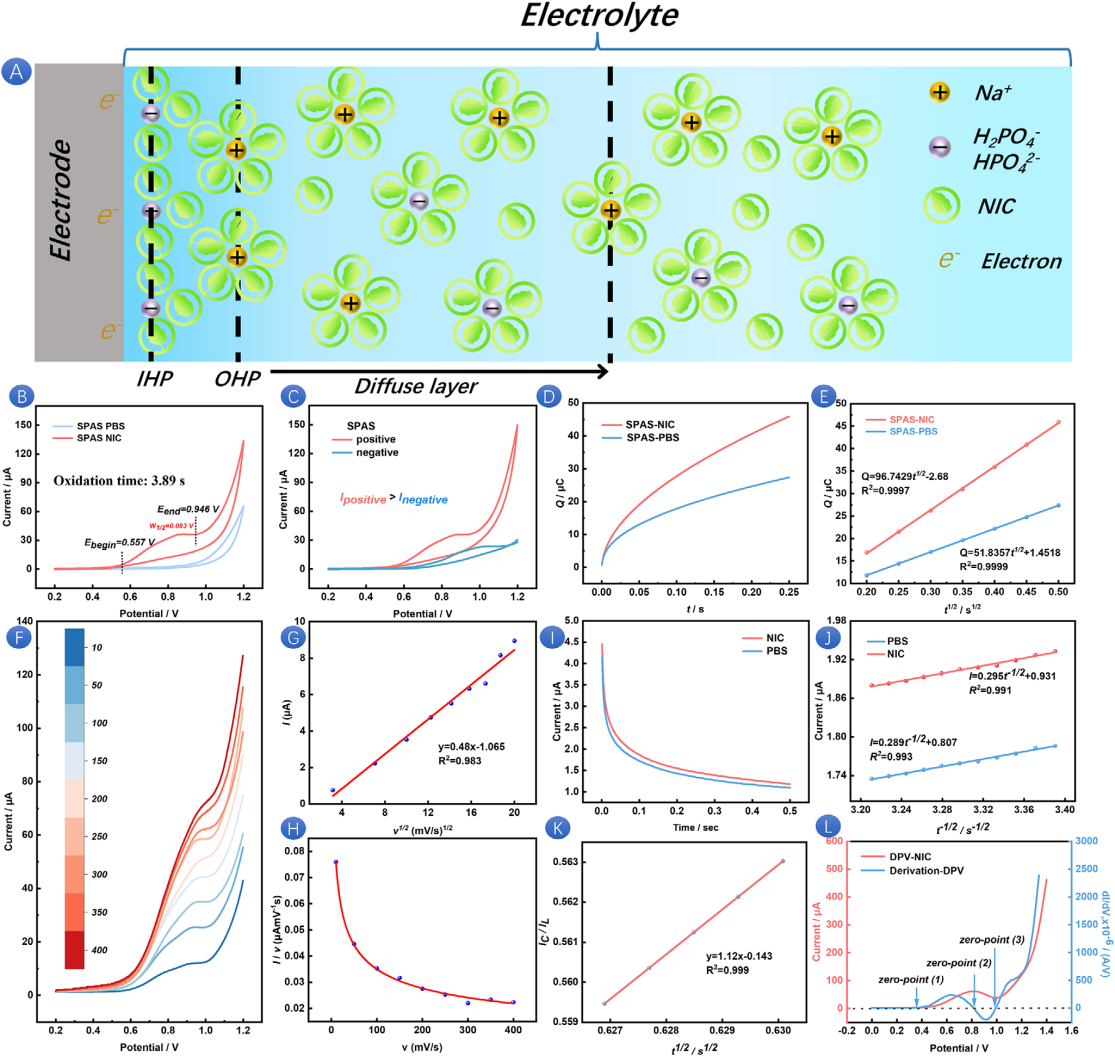
Electric double layer model of NIC in PBS (A). CV of SPAS in PBS (pH 8) with 500 µM NIC and without NIC (B). CV of SPAS with positive and negative scanning in PBS (pH 8) containing 500 µM NIC (C). Chronocoulometric curves of SPAS in PBS (pH 8) containing 500 µm NIC (D). *Q*–*t* ^1/2^ curves of SPAS (E). LSV curves of SPAS at different scanning rates in PBS (pH 8) containing 500 µM NIC (F). Relationship between peak current and *v* ^1/2^ (G). Normalized current (*I/v*) versus scanning rate curves of SPAS (H). Chronoamperometry curve of SPAS in PBS (pH 8) with 500 µmM NIC and without NIC (I). *I*–*T* ^−1/2^ curves of SPAS (J). *I*_C_ */ I*_L_–*t* ^1/2^ curves of SPAS (K). DPV curve of SPAS and the derivative (*dI/dV*) diagram of the DPV curve in PBS (pH 8) containing 500 µM NIC (L).

When the SPAS comes into contact with a buffer solution containing NIC, a typical double‐layer structure spontaneously forms on the electrode surface. Under the driving force of an external potential, the surface electrons of the electrode undergo directional migration with electrolyte ions, leading to the phenomenon of interface charge rearrangement. Excessive ion charges accumulate near the electrode surface in the electrolyte solution. The structure formed by these directional dipoles and charged materials can be conceptualized as two parallel layers, as shown in Figure 1A. The layer closest to the electrode, commonly known as the Helmholtz layer or Stern layer, is composed of solvent molecules, NIC, and occasionally specifically adsorbed substances. The specific adsorption ions HPO_4_^2−^ and H_2_PO_4_^−^, as well as the recent non‐specific adsorption ion Na^+^, form the “inner Helmholtz plane” (IHP) and “outer Helmholtz plane” (OHP), respectively. The second layer is a dynamic diffusion layer, whose ion distribution is synergistically regulated by long‐range Coulomb forces and thermodynamic disturbances, extending outward from OHP to the bulk solution phase. It is worth noting that the spatial arrangement of ions in the diffusion layer exhibits gradient decay characteristics, which is in sharp contrast to the disordered distribution of ions in the solution itself.

The electrocatalytic activity of NIC on SPAS was investigated using CV, as shown in Figure 1B. In the absence of NIC in the blank PBS buffer (pH 8), no irreversible oxidation peak is detectable in SPAS. However, upon the addition of 500 µM NIC to the PBS, a distinct oxidation peak emerges at a potential range of 0.557 to 0.946 V [34]; the oxidation time of NIC is 3.89 s at the scan rate of 0.1 V/s. The half peak width (*W*_1/2_) obtained is 0.083 V, indicating a fast electron transfer rate. The effect of positive and negative scanning on the CV peak current of NIC was examined, as illustrated in Figure 1C. It is found that the peak current for the positive scanning on SPAS was greater than that for the negative scanning, likely because oxidation reactions are more likely to occur under positive potential. The position and intensity of the oxidation peak, which can be ascertained through forward scanning, are crucial for understanding the kinetic characteristics of the electrochemical reaction. To clarify the electrochemical signal of NIC, the adsorption behavior of NIC on SPAS was further studied. Record chronocoulomb curve of SPAS in PBS (pH 8) containing 500 µM NIC (Figure 1D) and further convert it into the charge (*Q*) versus the square root of time (*t* ^1/2^) curve (Figure 1E) according to Anson equation (Equation 3) [29].

(3) $$Q\left(t\right)=\frac{2nFACD^{1/2}\;t^{1/2}}{\pi^{1/2}}+Q_{dl}+Q_{ads}$$

In the absence of any analyte, the interception of the equation corresponds to the charge of the electric double layer (*Q*_dl_). When NIC is present, the interceptive value represents the combined charge of the *Q*_dl_ and the charge adsorbed on NIC (*Q*_ads_). Based on the equation, the *Q*_dl_ and *Q*_ads_ for NIC on SPAS are determined to be 1.45 µC and 1.23 µC, respectively. These results indicate that SPAS is capable of effectively accumulating NIC. The effect of scanning rate on the linear sweep voltammetry (LSV) response of SPAS in PBS (pH 8) containing 500 µm NIC was investigated. As depicted in Figure 1F, an increase in the scanning rate leads to a corresponding increase in the LSV peak current value, with the peak potential shifting toward more positive values. These findings clearly indicate that the electrochemical reaction of NIC on SPAS is kinetically limited. Moreover, the linear relationship between the peak current and the square root of the scanning rate (*v* ^1/2^), as shown in Figure 1G, suggests that the oxidation process of NIC on the electrode surface in a weakly alkaline environment is diffusion‐controlled. Additionally, the relationship between the normalized current (*I/v*) and the scanning rate was explored to further understand the electrochemical oxidation of NIC on SPAS. The result presented in Figure 1H shows that the normalized current decreases with increasing scanning rate and then levels off, which is characteristic of a typical electrocatalytic (regenerative) EC mechanism [35]. In step chronoamperometry (CA), a step potential is repeatedly applied between the WE and the RE as the stimulus, with the pulse width of the electrode reaction at both the highest and lowest potentials being identical. The experimental outcomes are displayed in Figure 1I. Further analysis of this data yields the linear correlation curves of *I*–*T* ^−1/2^ (Figure 1J) and *I*_C_ */I*_L_–t^1/2^ (Figure 1K). The apparent diffusion coefficient (*D*) and the reaction rate constant (*K*) of NIC on SPAS can be estimated using Cottrell's equations [36] (Equation 4) and (Equation 5), respectively.

(4) $$I=nFAD^{\frac{1}{2}}c_{0}\pi^{-\frac{1}{2}}t^{-\frac{1}{2}}$$

(5) $$\frac{I_{C}}{I_{L}}=\pi^{\frac{1}{2}}{\left(Kc_{0}t\right)}^{\frac{1}{2}}$$

*N* is the number of electron transfer in the reaction, *F* is Faraday constant, *A* is the electroactive area of the electrode, *C_0_* is the initial molar concentration, *T* is the electrolysis time, *I*_C_ is the oxidation current of NIC in PBS, *I*_L_ is the limiting current of PBS. Therefore, the *D* value of NIC on SPAS is calculated as 0.82 × 10^−14^ cm^2^/s, and *K* is 1.6 × 10^−6^ L/(µM·s). The larger apparent coefficient indicates that NIC is affected by interface charge transfer on SPAS and has a good electrochemical reaction rate.

We utilized differential pulse voltammetry (DPV) to conduct a preliminary exploration of 500 µm NIC. Concurrently, the derivative of the DPV curve was processed to detect any discontinuities within the curve, which can provide insights into the electrochemical behavior of NIC [37]. As shown in Figure 1L, the first derivative spectrum of the DPV curve exhibits three characteristic zeros, corresponding to the key inflection points of the original voltampere curve. Specifically, after the first zero point, the positive range of the derivative spectrum (*dI/dV*>0) characterizes the monotonic increase trend of the DPV current, corresponding to the beginning oxidation stage of the NIC oxidation reaction. When the potential scan reaches the second zero point, the DPV response current exhibits a peak characteristic, indicating that the kinetic maximum of the NIC oxidation reaction rate is at the electrode surface. Subsequently, the derivative spectrum changed from positive to negative (*dI/dV*<0), indicating that the reaction entered the diffusion‐controlled decay stage, and the mass transfer limitation of active substances in the system led to a gradual decrease in current intensity. Finally, when the potential crosses the third zero point, the DPV current tends to a steady state, confirming the completion of the NIC oxidation reaction. The chemical signal is completely converted into a detectable electrochemical signal through the Faraday electron transfer process.

In order to explore the electrochemical behavior of NIC in different pH environments and different scanning rates, the effect of scanning rate on the electrochemical oxidation of NIC on SAPS was measured by CV in different pH PBS (pH 6, 7, 9). An irreversible oxidation peak of NIC is observed in the CV diagram at three different pH values, and *I*_pa_ increases with the increase of scanning rate, and the potential shifts slightly to the positive direction (Figure S2A,E,I), which is an irreversible electrode reaction driven by adsorption [38]. From Figure S2C, G, K and S2D, H, L, the linear relationship between *I*_pa_ and *v* and the linear relationship between *I*_pa_ and *v* ^1/2^ at three different pH values can be obtained, indicating that NIC is controlled by adsorption and diffusion relatively simultaneously on the electrode surface, but diffusion control is the main control [39]. According to Figure S2B,F,J, the linear regression curve of the natural logarithm of the anodic peak potential versus the scanning rate is obtained. It is found that the effect of three pH values on electrochemical kinetics is small. According to Laviron's theory, the relationship between *E*_pa_ and *lnv* is as follows (Equation 6) [40].

(6) $$E_{ox}=E^{0}+\left(\frac{RT}{\alpha nF}\right)\ln\left(\frac{RTK^{0}}{\alpha nF}\right)+\left(\frac{RT}{\alpha nF}\right)\ln v$$

Where *α* is the transfer coefficient, generally 0.5, *K^0^* is the apparent rate constant of surface reaction, *v* is the scanning rate, *n* is the number of electron transfers, *R*, *T*, and *F* have been proposed in Equation 6. The slope of the curve is equal to *RT/αnF*. Therefore, the electron transfer number of NIC oxidation on the electrode surface at three different pH values is calculated to be 2. It is worth noting that two oxidation peaks can be observed in pH 9 (Figure S2I), but not in acidic and neutral environments. This is due to the formation of the NIC free radical intermediate and the removal of an electron from neutral NIC species [41]. The frontier molecular orbital theory divides the molecular orbital into the highest occupied molecular orbital (HOMO) and the lowest unoccupied molecular orbital (LUMO). The smaller the frontier orbital, the harder it is to excite the electrons, and the more energy is required for them to participate in chemical reactions. To verify the formation process of the intermediate shown in Figure S3A and the possibility of secondary redox reactions, we calculated the HOMO–LUMO (Figure S4) and spin isodensity of the intermediate, and compared it with NIC. As shown in Figure S3B, compared with NIC, the intermediate exhibits a significant Spin isodensity surface, indicating that NIC experienced the loss of one electron and one proton in the initial reaction step, resulting in the formation of the intermediate [42]. Further from Figure S3C, the HOMO energy level of the NIC is higher than that of the intermediate, indicating that the oxidation potential required for the HOMO electronic transition of the NIC is lower than that of the intermediate. This calculation result is consistent with experimental observations, that is, the oxidation potential of the intermediate is higher than that of NIC, thus supporting the rationality of the secondary redox process.

**FIGURE 2:**
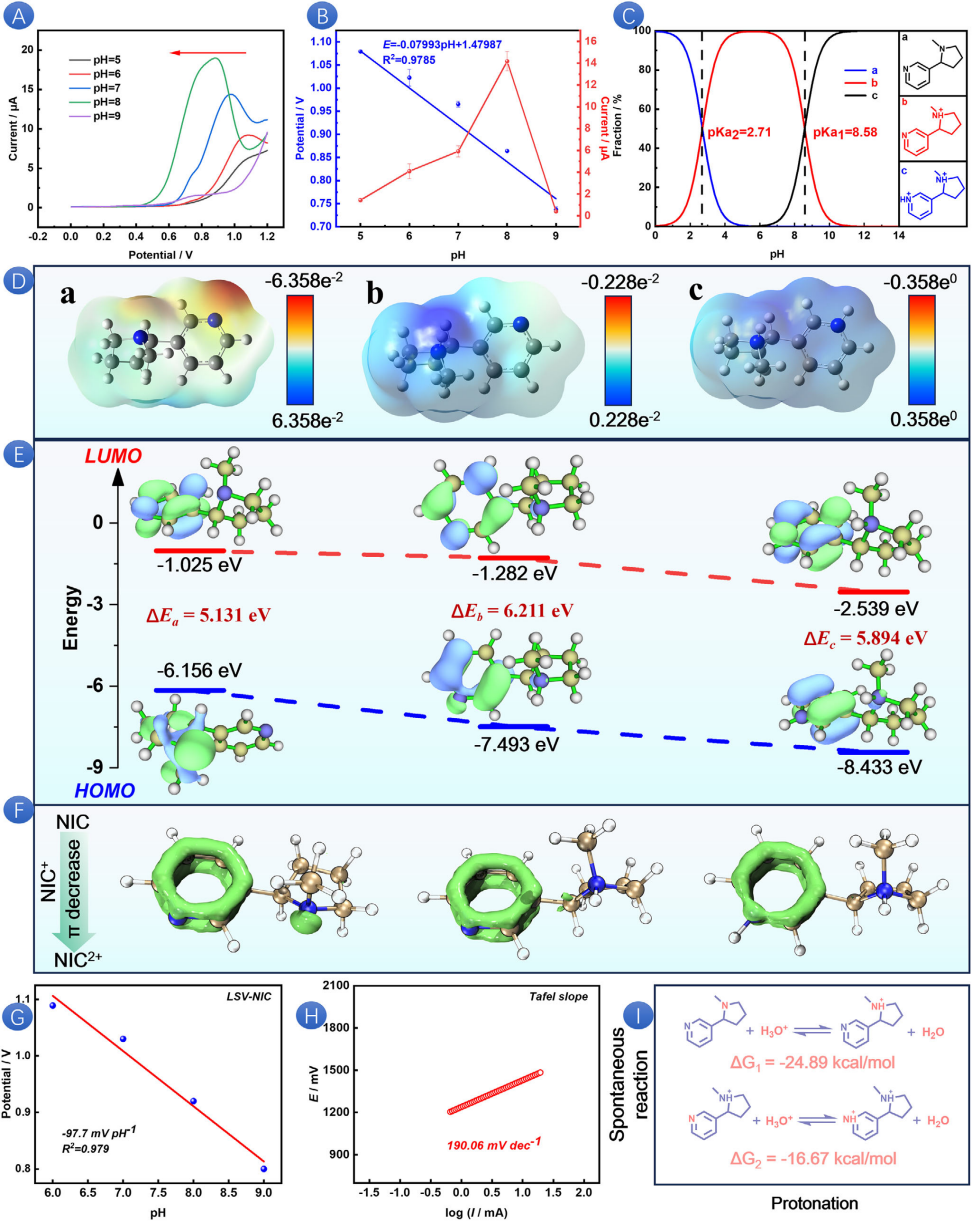
DPV curve (A) of SPAS in PBS containing 200 µM NIC with different pH (5∼9). Linear relationship (B) between *E*_pa_ or *I*_pa_ and pH. Morphological fitting of NIC at different pH (C). ESP (D) of NIC (a), NIC^+^(b), and NIC^2+^ (c). HOMO–LUMO calculations (E) of NIC, single‐proton NIC, and double‐proton NIC. LOL‐π isosurfaces (F) of NIC, NIC^+^, and NIC^2+^. Linear relationship between pH and *E*_pa_ (G). Tafel slope of NIC on SPAS (H). Gibbs free energy of NIC protonation process (I).

First, the effect of pH value on the current response of NIC was studied using DPV within the pH range of 5.0–9.0; the results are shown in Figure 2A. It is observed that the peak potential shifts slightly toward the negative direction as the pH increases, suggesting that protons are involved in the electrode reaction. The correlation between pH and both *E*_pa_ and *I*_pa_ is illustrated in Figure 2B. The current response reaches its maximum at pH 8, and the following equation is derived, *E_pa_* = −0.07993pH+1.47987 (R^2^ = 0.9785). When pH is equal to 0, the oxidation potential is at a very high position (1.48 V), which may have exceeded the potential window of the solution/electrode and led to hydrogen evolution. Therefore, NIC electrooxidation needs to be carried out in a reasonable buffer solution. The slope of this relationship is close to the theoretical value, which implies that the number of protons participating in the NIC oxidation is equivalent to the number of electrons transferred in the reaction. The NIC electrooxidation involves the participation of two electrons and two protons [43]. Then the interaction model between NIC and H_2_O is simulated, and the results are shown in Figure S5. It is worth noting that the two N atoms on NIC are easy to combine with the H atom on H_2_O through coordination bonds, so it is speculated that NIC is prone to protonation to varying degrees in different pH environments. As depicted in Figure S6, the initial protonation of NIC can occur via two distinct pathways, either on the pyridine ring through an sp^2^ hybridized orbital or on the pyrrole ring through an sp^3^ hybridized orbital. Next, we elucidate the initial protonation site of NIC by calculating the energy required for NIC and H‐bond breaking. The results are presented in Figure S7. As shown in Figure S7A, the energy required is higher than that in Figure S7B, indicating that the former structure is more stable than the latter. This suggests that protonation preferentially occurs on the pyrrole ring, followed by the completion of biprotonation on the pyridine ring. Figure S7C illustrates the energy required for the fracture of the H‐bond in the second protonated state. To further elucidate the effect of pH on the oxidation of NIC, we performed a morphological fitting analysis [44] of NIC (Figure 2C). The proportions of NIC forms in the pH range of 5–9 are shown in Table S1. NIC behaves as a weak biacidic base, exhibiting two pKa values pKa_1_ = 8.58 and pKa_2_ = 2.71. These values correspond to the protonation of the pyridine nitrogen in the NIC molecule, with pKa_1_ representing the monoprotonated form and pKa_2_ indicating the biprotonated form. In the pH range of 0–6, NIC is unstable, and there is a mixture of monoprotons and biprotons. Between pH 6 and pH 12, biprotonation of NIC does not occur, and only the monoprotonated form and a mixture of molecular species are present. The pKa_1_ value can be attributed to the partial acid–base functionality of the pyrrolidine moiety. When the pH is equal to or greater than 8.58, the non‐protonated free base form of NIC predominates in the electrolyte. This pH‐dependent behavior significantly influences the electrochemical properties and reactivity of NIC. The relationship between oxidation potential, oxidation current, and pH value is complex, and further research is needed to fully understand the potential mechanism.

Electrostatic potential (ESP) plays a crucial role in probing electrostatic interactions between molecules, pinpointing reactive sites, and forecasting molecular properties [45]. ESP further clarifies the protonation effect of NIC. As shown in Figure 2D, the electrostatic potential value of the two N atom regions of NIC are more negative, so NIC has good electron donor performance and is easy to be electrochemically oxidized; It is worth noting that with the protonation process of NIC, the electrostatic potential value at the two N atoms increases, so it is less and less vulnerable to electrophilic attack. The Fukui function is applicable to various reactions, including electrophilic reaction, nucleophilic reaction, and free radical reaction. This versatility enables us to have a subtle understanding of how different types of chemical reactions interact with a given molecular structure. According to this theory, the larger the *f^+^*, the easier it is to be attacked by nucleophiles, and the larger the *f* ^−^, the easier it is to be attacked by electrophiles [46]. The calculation results are presented in Figure S8. It can be observed that the *f* ^−^ value ofthe two N atoms decreases with the progress of the protonation process. This indicates that the likelihood of NIC being subjected to electrophilic attack diminishes during this process, which is in line with our ESP calculation results. The lone pair electrons on N show strong electrophilicity after binding with protons through coordination bonds, which improves the oxidation resistance of NIC in the protonation process. Therefore, when the pH value is less than 5, the electrochemical behavior of NIC decreases significantly. The potential shifts to the negative direction with the decrease of pH and protonation according to the pH value of the solution. It is worth noting that with the protonation process of NIC molecules, the energy values of HOMO and LUMO have gradually decreased (Figure 2E). At the same time, we calculated HOMO–LUMO energy gap of NIC, NIC^+^, and NIC^2+^ in the vacuum state. The results are shown in Figure S9, and the same phenomenon can be observed. The change of molecular orbital energy is due to the change of electron absorption of functional groups [47]. By delocalizing the π electrons of the molecule to the entire molecular plane, as shown in the Localized Orbital Locator–π (LOL‐π) isosurface of Figure 2F, the delocalization of the π electrons in the entire structure decreases with the protonation of the NIC. This reduced molecular conjugation effect leads to a decrease in the HOMO–LUMO orbital, which is consistent with the calculated results mentioned above [48]. When pH<pKa, the solution gradually transforms into cationic form and gradually shows a lower reaction tendency. The harder it is to excite electrons, the higher the required potential. Therefore, the peak current shifts in the positive direction as the pH decreases.

The LSV curve presented in Figure S10A illustrates that the oxidation potential of NIC is contingent upon the pH of the electrolyte. By fitting the linear relationship between pH and the oxidation potential (Figure 2G), we determine a slope of −97.7 mV/pH. At the same time, as shown in Figure S10B, the same rule can be observed that the initial oxidation potential gradually decreases with the increase of pH. This finding indicates that the electrooxidation of NIC is characterized by a PCET mechanism. To delve deeper into the kinetics of this PCET process, the Tafel slope analysis can be utilized, offering a means to explore the kinetic parameters and shed light on the interplay between protons and electrons in the electrochemical oxidation of NIC [49]. As illustrated in Figure 2H, the Tafel slope for the electrooxidation of NIC is measured to be 190.06 mV/dec. The electron transfer coefficient (*α*) can be derived using Equation 7. Considering the influence of pH, the calculated electron transfer coefficient α is found to be 0.16 (<0.5). This is associated with the protonation state of NIC, particularly during the pH reduction process, suggesting that the protonation dynamics play a significant role in the rate‐determining step of the electrochemical reaction. Therefore, the reaction order of proton activity is calculated by Equation 8 [50], the reaction order of NIC in the protonation process is 0.51, which may indicate that there is a zero‐order reaction independent of NIC concentration in [H^+^]. To substantiate the aforementioned hypothesis, we employed computational chemistry to determine the thermodynamic parameters associated with the protonation of NIC, as detailed in Table S2. The Gibbs free energy change (Δ*G*) for the monoprotonation process is calculated to be −29.36 kcal/mol (Δ*G*<0), while for the biprotonation process, it is −17.68 kcal/mol (Δ*G*<0) (Figure 2I). At the same time, the enthalpy changes in the process of monoprotonation and biprotonation can be calculated, which are −24.89 kcal/mol and −16.67 kcal/mol (Δ*H*<0), respectively. These thermodynamic parameters confirm that NIC exhibits significant self‐heating characteristics in both protonation pathways, and the single protonation pathway has a better thermodynamic driving force, promoting the system to evolve toward a more stable protonation state. Continuing with the discussion on the solvent recombination energy of NIC protonation [51], Equation 9 can be used to calculate the solvent recombination energies of single protonation and biprotonation, which are 1.18eV and 2.67eV, respectively. The relative ratio indicates that when the second proton is added to a positively charged molecule, it needs to overcome the huge solvent recombination energy cost and intramolecular electrostatic repulsion. Consequently, the core position of the intrinsic protonation effect of molecules in NIC electrooxidation has been established. In terms of reaction kinetics regulation under different pH conditions, the protonation process not only affects the surface adsorption configuration of reactants but also significantly regulates the overall reaction rate by changing the charge transfer pathway.

(7) $$b=\frac{2.3RT}{\propto nF}$$

(8) $$\frac{\partial E}{\partial pH}=-\left(\frac{\partial E}{\partial logI}\right)\left(\frac{\partial logI}{\partial pH}\right)$$

(9) $$\lambda =\frac{1}{2}\left|\Delta G_{solv,initial}-\Delta G_{solv,final}\right|$$

### 2.3 NIC Oxidation Mechanism Under Acid and Alkali Conditions

**FIGURE 3:**
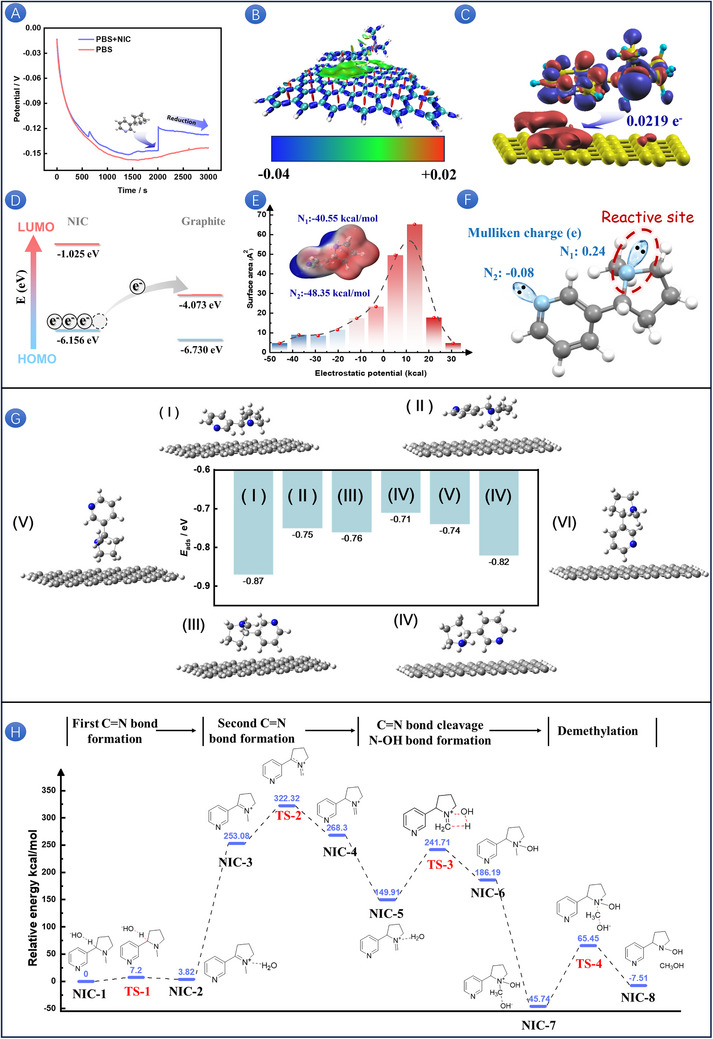
Open circuit potential versus time curve of SPAS in PBS (pH 8) with 500 µm NIC and without NIC (A). Weak interaction between NIC and graphite (B). Differential charge density between NIC and graphite (C). Schematic diagram of orbital electron transitions in NIC (D). Bar chart of the distribution of electrostatic potential values on the surface area of NIC (E). Mulliken charge of two N atoms in NIC (F). Structural configurations and energies of NIC adsorbed on graphene with different orientations (G). Gibbs free energy spectrum transition state of the NIC electrooxidation process under neutral and alkaline conditions (H).

The complexity of the interface reaction mechanism and oxidation pathway on the graphite surface significantly limits the in‐depth analysis of the dynamic behavior of the process in the electrochemical oxidation of NIC driven by double proton double electron coupling. As shown in Figure 3A, through the analysis of the curve of open circuit potential (OCP) versus time, it is found that the introduction of the NIC molecule significantly changes the interface characteristics of the electrooxidation system. The adsorption process of NIC molecules on the graphite surface caused a positive shift in the open circuit potential at 2000 s, and the subsequent electric potential relaxation phenomenon (T>2000 s) can be attributed to the dynamic evolution of the interface electron transfer mechanism [52]. As shown in Figure 3G, we calculated the adsorption energy of NIC on graphite and placed NIC on the graphite surface, orientation adsorption of the six best possible pathways. Among them, structure I has the lowest energy as the most likely orientation structure, so it is selected as the research object for subsequent interface mechanisms, and other results will not be further studied [53, 54]. This adsorption process belongs to the spontaneous behavior driven by heat release. There is an interaction force between NIC and graphite, which makes the entire system more stable. It is speculated that the possible interaction force is van der Waals force. To prove the above conjecture, we calculated the weak interaction between NIC and graphite (Figure 3B). The green between the two represents the van der Waals interaction region. A smaller steric hindrance is more conducive to the transition of orbital electrons on NIC [55]. As shown in Figure 3C, after adsorption, the electron density of NIC molecules decreases as an electron donor, while the electron density of the graphite layer increases as an electron acceptor, with a transferred charge value of 0.0219 e^−^ [56]. This discovery indicates the electron transfer mechanism of NIC to the graphite surface. When a certain potential is given to the structure, as shown in Figure 3D, the HOMO orbital electrons of NIC begin to transition to the LUMO orbital of graphite, and density functional theory (DFT) calculation confirms the transfer of NIC electrons from the perspective of energy [57, 58]. To systematically analyze the initial active sites of the electrooxidation reaction of the NIC molecule, the quantitative analysis of electrostatic potential based on Figure 3E shows that the interactions between NIC molecules are mainly dominated by electrostatic forces, which can be attributed to the stable hydrogen bonding network formed between their molecules. The nitrogen atoms of pyrrole ring (N_1_) and pyridine ring (N_2_) exhibit similar electrostatic potential values due to their lone pair electron distribution characteristics, suggesting that these two sites may become potential starting points for electrooxidation reactions. Subsequently, the Mulliken charges [59] of two N atoms are calculated, and as shown in Figure 3F, N_1_ is positively charged, resulting in a significant decrease in electron density compared to its isolated atomic state. This led to a decrease in its effective electronegativity, making it more susceptible to electron loss during electrochemical oxidation. It is worth noting that N_2_ is negatively charged, and its higher electron density can be attributed to the stable delocalized π‐electron structure formed by its hexagonal aromatic ring system. Therefore, the electrooxidation reaction of the NIC molecule preferentially begins at N_1_.

Based on the research, we proposed different oxidation processes under acidic and alkaline conditions in Figure S11. In the key step of the alkaline oxidation reaction, as shown in Figure S12, there is a significant difference in the energy barrier between the first step dehydrogenation (bond breaking energy: 363.22 kcal/mol) and the fourth step demethylation (bond breaking energy: 43.86 kcal/mol), indicating that the former is the rate‐determining step. In addition, we proposed PCET steps for the electrooxidation process under different conditions (pH 5–9) in Figure S13. And due to its non‐covalent adsorption properties, there is no strengthened chemical bond formed between NIC and the graphite electrode, which belongs to the typical Outer‐sphere PCET [60]. The transfer of electrons and protons can be achieved through distributed or coordinated pathways, depending on the pH conditions of the system. In this process, the electrode acts as an inert electron donor or acceptor and does not directly participate in the formation and cleavage of chemical bonds. The key point is that proton transfer is driven by the process of electron tunneling; electrons pass through the electrochemical double layer (EDL) and reach outside the OHP, where they are accepted by the molecular active centers in a bound or freely diffusing state, thereby inducing proton migration. Based on the above research, the PCET process of NIC can be regarded as a multistep collaborative process spanning multiple adiabatic potential energy surface minima under the joint action of solvent coordinates and outer sphere configuration coordinates [61]. The transition state of the complex reaction path under alkaline conditions (the best detection condition) was discussed. The system elucidates the multilevel synergistic mechanism of NIC oxidation reaction under alkaline conditions and reveals the dynamic evolution process mediated by hydroxyl groups. As shown in Figure 3H, during the formation of the first C═N double bond, NIC first reacts with the hydroxyl group to form the intermediate NIC‐1. Subsequently, the hydroxyl nucleophile attacks the H atom on the C atom, producing water molecules that are further converted into the intermediate NIC‐2. At this point, the C═N bond begins to form, accompanied by the accumulation of positive charges on the N atom. The increase and decrease of energy during this process are accompanied by the breaking of C─H bonds and the formation of O─H bonds. After removing water molecules, the stability of the overall structure is disrupted, resulting in an increase in Gibbs free energy. In the second stage of C═N double bond formation, the transition from NIC‐3 to NIC‐4 involves intramolecular hydrogen transfer, achieving double bond rearrangement while maintaining the positive charge state of the N atom. Then NIC‐4 reacts with water molecules to form intermediate NIC‐5, which promotes structural stabilization through water molecules. Under the action of water addition, the C═N double bond is broken, accompanied by the production of hydroxyl groups exhibiting endothermic characteristics, forming NIC‐6. In the final demethylation stage, NIC‐6 reacts with OH^−^ to form the intermediate NIC‐7. Hydroxyl groups induce methanol elimination through intermolecular interactions, completing the electronic reconstruction of aromatic systems and obtaining the final oxidation product NIC‐8. The entire oxidation process of NIC exhibits exothermic characteristics, and the system achieves a reduction in free energy through gradual bond recombination. The study reveals that water molecules undertake a proton shuttle function, and hydroxyl groups dominate the reaction process through charge stability and transition state stabilization, providing atomic‐scale kinetic analysis for alkaline oxidation mechanisms.

### 2.4 Molecular Dynamics Simulation of NIC on SPAS Portable Sensor

**FIGURE 4:**
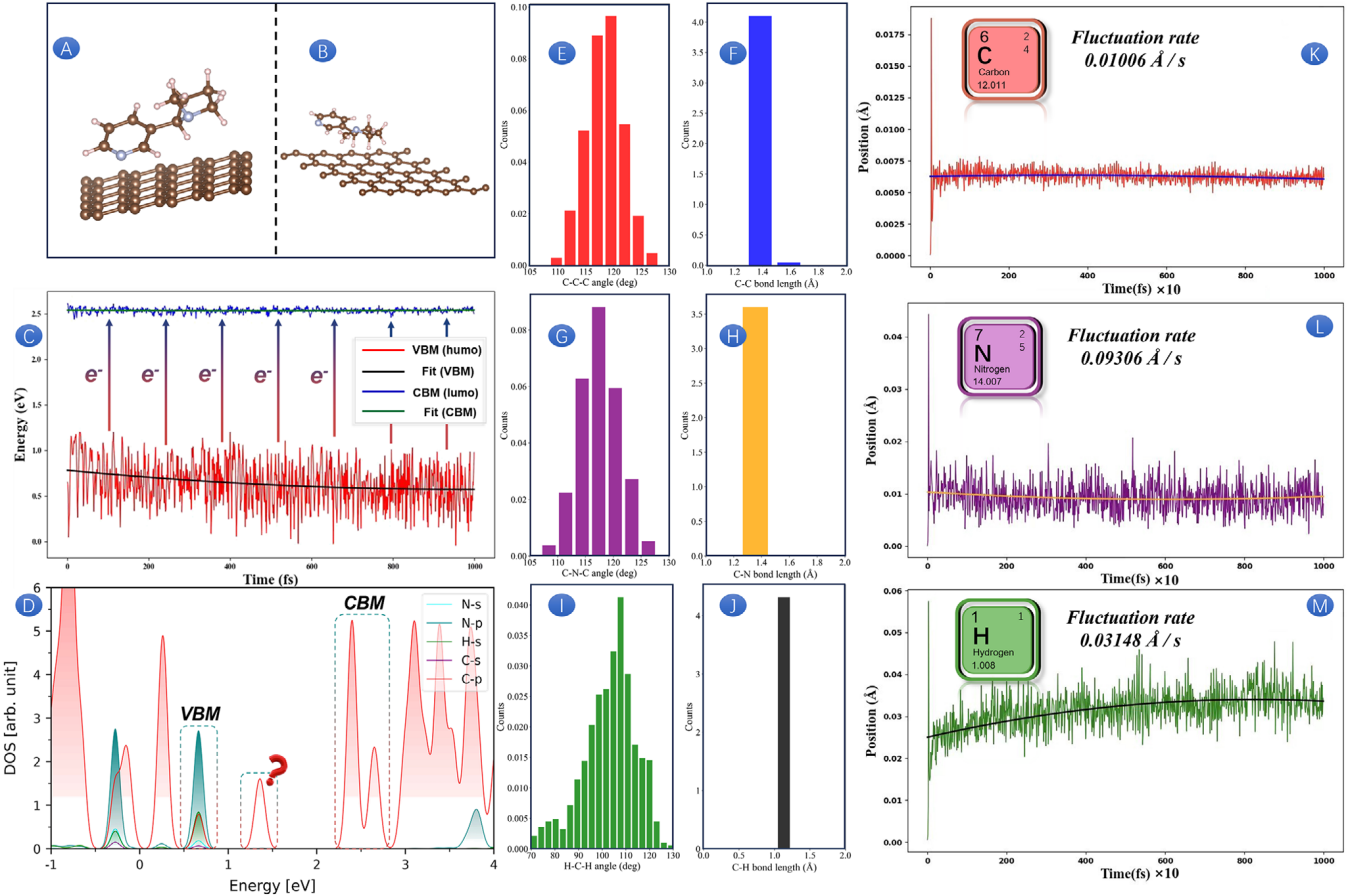
NIC and the optimized structure of graphite (A). Average structure of NIC and graphite MD simulation (B). Energy level fluctuation diagram (C) based on NIC and graphite. Density of states analysis based on NIC and graphite (D). Change of C─C─C bond angle (E). Change of C─C bond length (F). Change of C─N─C bond angle (G). Change of C─N bond length (H). Change of H─C─H bond angle (I). Change of C─H bond length (J). Fluctuation rate of C atom (K). Fluctuation rate of N atom (L). Fluctuation rate of C atom (H).

To verify the argument of the phenomenon‐conformation‐mechanism mentioned above, as well as the stability of NIC at the sensing interface, the micro dynamic process of dynamics was used to explain. The initial step in this analysis is to optimize the molecular structures of both graphite and NIC, as illustrated in Figure 4A. Figure 4B shows the average structure of a 5 Ps adiabatic Molecular Dynamics (MD) simulation at 300 K. The MD simulation results, detailing the evolution of molecular orbital energies, are presented in Figure 4C. It is observed that the energy level fluctuations of the conduction band (CBM) are considerably smaller compared to those of the valence band (VBM). This suggests that the VBM is more susceptible to external influences, facilitating the excitation of electrons to transition to the CBM. This finding is corroborated by the statistical analysis of bond angles for C─C─C, C─N─C, and H─C─H, as well as bond lengths for C─C, C─N, and C─H, as depicted in Figure 4E–J. Subsequent calculations reveal that the fluctuation rates of the C, N, and H atoms are 0.01006, 0.09306, and 0.03148 Å, respectively, as shown in Figure 4K–M. The C atom's fluctuation rate is significantly lower than that of the N and H atoms, which can be attributed to the stable structure of graphite. Given the minimal fluctuation amplitude of the C atom, it is hypothesized that the CBM energy level in this calculation is predominantly contributed to by the C atom. Conversely, the substantial fluctuation amplitudes of the N and H atoms suggest that they are the primary contributors to the VBM energy level. To validate this hypothesis, a calculation and analysis of the density of states (DOS) was conducted, as presented in Figure 4D. The results provide a clear visual representation that the N and H atoms contribute to the VBM energy level, while the C atom is responsible for the CBM energy level. The DOS analysis reveals an asymmetry, with the DOS at the VBM being lower than that at the CBM, suggesting that the state distribution at the CBM is more concentrated [62], which is mainly contributed by the C atom in graphite. An intriguing discovery is the presence of a defect state within the band gap between the CBM and VBM. Given that this state is entirely contributed by C atoms, it is preliminarily hypothesized to be a metastable defect state arising from the graphite molecules. This defect‐enriched graphite coating is also believed to play a crucial role in the electrooxidation of NIC [63]. As mentioned above, the molecular dynamics behavior of NIC on SPAS confirms that both the electrooxidation process and protonation behavior are related to N and H atoms, and NIC can remain stable throughout the entire electrochemical response process.

### 2.5 Multi‐Scenario NIC Detection on SPAS Portable Sensor

**FIGURE 5:**
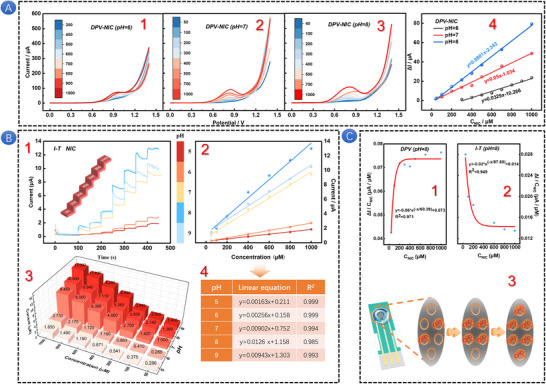
DPV curves of SPAS in PBS containing different concentrations of NIC at pH 6–8 (A1–A3). Linear curve of NIC concentration and *I*_pa_ at pH 6–8 (A4). I–T curve of NIC and the linear curve (B1‐B2) of current and corresponding NIC concentrations at pH 5–9. 3D histogram of NIC current changing with concentration at five different pH values (B3). Equation and correlation coefficient of I–T linear curve (B4). Nonlinear relationship between *I/c* and *c* of DPV and I–T at pH 8 (C1‐C2). Schematic diagram of NIC reaching the upper limit of detection (C3).

Figure 5A1–A3 shows the DPV response of NIC with different concentrations at three different pH values [64]. The linear relationship between peak current and concentration is shown inFigure 5A4. The peak current is proportional to NIC concentration in the linear range of 300–1000 µm at pH 6.0, 50–1000 µm at pH 7.0, and 40–1000 µM at pH 8.0. The sensitivity (*S*), detection limit (*LOD*), and limit of quantitation (*LOQ*) are calculated by the following Equation 10.

(10) S=m/ALOD=3s/mLOQ=10s/m

*A* is the geometric area of the working electrode (12.56 mm^2^), *s* is the standard deviation of the peak current of the lowest concentration within the linear range, and *m* is the slope of the relevant calibration equation. According to the calculation, the *S*, *LOD*, and *LOQ* in DPV mode are shown in Table S3. The *S*, *LOD*, and *LOQ* based on SPAS under alkaline conditions are better than those under neutral and acidic conditions, which is related to the pH‐controlled voltammetric behavior of NIC.

Figure 5B1,B2 shows the Chronoamperometry method (I–T) response of NIC with different concentrations at five different pH values and the linear relationship curve between the concentration and the corresponding current. Figure 5B4 shows the specific equation and relevant fitting degree. The *S*, *LOD*, and *LOQ* are further calculated by Equation 10. The *S*, *LOD*, and *LOQ* in I–T mode are shown in Table S3. From the 3D histogram of Figure 5B3, it can be concluded that the I–T detection mode has a wider pH detection range, and the detection performance in alkaline conditions is also greater than that in neutral and acidic conditions, which is also related to the dominant non‐protonated free base form of NIC in the electrolyte.

To ascertain that NIC has reached the upper limit of detection in both DPV and I–T methods, the detection results at pH 8 were selected for further analysis. The relationship between the current ratio and the corresponding NIC concentration was subjected to nonlinear fitting with respect to NIC concentration. This fitting process aids in elucidating the intricate relationship between the current response and NIC concentration, offering a mathematical model to comprehend the detection mechanism. As depicted in Figure 5C1,C2, the ratio of current to the corresponding NIC concentration exhibits an increase in the DPV method, eventually stabilizing, and a decrease in the I–T method, also eventually stabilizing. This behavior is attributed to the active sites on the working electrode being continuously occupied by the NIC molecule and undergoing oxidation. Due to the irreversible nature of the process, these active sites do not get freed up. Consequently, as the NIC concentration increases, the active sites on the electrode gradually become saturated, as illustrated in Figure 5C3, indicating that the detection has reached its upper limit.

We have conducted a comparative analysis of the detection limits and other characteristics of the two detection methods. In the DPV detection mode, the sensor demonstrates high sensitivity and a low detection limit. This is attributed to the reduction of background currents, such as the electric double‐layer charging current and impurity Faraday currents, among others. In contrast, the I–T detection mode offers a broader linear range and a more extensive detection environment across different pH levels. This may be a result of rapid signal acquisition, minimal pre‐electrolysis, and effective subtraction of capacitive background noise, which together contribute to the enhanced performance of the sensor in this mode [65]. Under optimal conditions, the two methods have similar linear ranges, indicating the quantitative reliability of both methods in detecting NIC. In addition, to verify the accuracy of the portable electrochemical sensor based on SPAS, it was corrected by UV–vis (Figure S14). The absorption peak at 260nm mainly comes from the pyridine ring of the NIC molecular structure. When absorbing ultraviolet light, electrons undergo a transition from π bonding orbitals to π* antibonding orbitals. The absorption peak at 210–220 nm is mainly related to the tertiary nitrogen atom of the pyrrolidine moiety. When absorbing ultraviolet light, lone pair electrons mainly transition to the π* antibonding orbitals of the pyridine ring and may also transition to the σ* antibonding orbitals of the C─N or C─C bonds. Next, the analytical performance of the proposed portable electrochemical sensor is compared with the previously reported NIC sensor, and the results show that the proposed method can be applied to the detection of NIC (Table S4).

Before each DPV measurement, the SPAS was pretreated with five cycles of CV (Figure S15A–D), and four different potential ranges were set. The first solid line is not pretreated by CV. By increasing the detection times under the same conditions, the continuous DPV curves are almost completely coincident, indicating that SPAS has good anti‐pollution ability. We added 10 times the concentration of substances (Dopamine, Glucose, and Cotinine) and 100 times the concentration of substances (CaCl_2_, FeCl_3_, KCl, MgCl_2_, NaCl, ZnCl_2_) to PBS (pH 8.0) containing 500 µM NIC, respectively, and then measured the DPV peak current value of NIC at SPAS. As shown in Figure S15E, there is no significant difference in NIC DPV peak current even when the above interfering substances exist. Based on the DPV peak current value of 500 µM NIC detected by ten independent SPAS under the same conditions (Figure S15F), the results show that there is no significant difference. Similarly, 500 µM NIC is detected at a fixed interval of one month, and SPAS is maintained at 4°C after each measurement, which can maintain a stable current signal during the storage time of up to a year (Figure S15G). Finally, the repeatability of SPAS was investigated. After 5 times of repeated detection of the same SPAS, it is found that the signal current gradually decreased, which is attributed to the irreversible oxidation reaction of NIC, indicating that the SPAS is a one‐time analysis strip (Figure S15H).

To assess the efficacy of the portable electrochemical sensor in practical applications, we collected NIC samples from four distinct scenarios: tobacco leaves from the field, dried tobacco leaves, cigarettes, and the saliva of individuals with a history of smoking. Under the optimized detection conditions, a known concentration of standard NIC was added into actual sample solution, and electrochemical detection was performed using DPV at SPAS sensors. Figure S16 presents the NIC concentration in these four types of samples as determined by the SPAS portable sensor. The results are in good agreement with the recovery rates of NIC, which range from 98.34% to 102.77%, indicating the high accuracy and reliability of the sensor in real scenarios. Meanwhile, the sensor is used for in vivo detection of NIC in live tobacco leaves. The test results are shown in Figure S17. The SPAS portable sensor not only shows the advantages of diversification in the traditional static in vitro NIC detection but also reflects the advantages of portability and rapidity in the dynamic in vivo NIC detection.

## 3 Conclusion

The electrooxidation mechanism of NIC at different pH levels was proposed on SPAS. The behavior of the NIC molecule on the graphite layer was simulated by molecular dynamics, and the information on microscopic energy levels between the interface and signal molecule was decoded. The proton dissociation equilibrium of NIC and the theoretical calculation model were used to explain the voltammetric behavior of peak potential and peak current, which were easily affected by pH in the process of NIC electrooxidation. The findings demonstrate that the oxidation of NIC on SPAS proceeds via an irreversible mechanism, predominantly governed by diffusion with a secondary contribution from adsorption. The overall process exhibits significant pH dependence (pH 5–9), underscoring the necessity of considering pH effects in investigations of the electrochemical oxidation of NIC. DFT calculations provide theoretical support for the interaction between NIC and the sensing interface, while elucidating the weakening of oxidation ability during the protonation process of NIC. These calculations highlight the importance of van der Waals forces and electron transfer in mediating the interaction between NIC and sensor surfaces. Applying classical transition state theory to the electrooxidation reaction of NIC at the sensing interface provides a new research perspective. In DPV detection mode, the detection limit, sensitivity, and detection range of NIC in alkaline (pH 8) conditions were better than those of acidic (pH 6) and neutral (pH 7) conditions. Under I–T detection mode, the detection limit and sensitivity of NIC at pH 8 are better than those under other conditions (pH 5, 6, 7, 9), and both methods have more significant sensing performance under alkaline conditions. The developed sensor demonstrates remarkable anti‐interference capability (selectivity coefficient >9.8), long‐term stability (12‐month performance retention>94%), and batch reproducibility (RSD<3.6%). Practical applications were successfully quantified NIC in diverse scenarios, such as fresh tobacco leaves, cured leaves, cigarettes, and smokers' saliva. It provides practical and reliable insights for the development of modern and information‐based NIC electrochemical sensors and provides an original way of thinking in the cognition of NIC electrooxidation. It has rigorous theoretical guidance and reliable practical reference values for NIC in agriculture, medicine, scientific research, and other fields.

## 4 Experimental Section

### 4.1 Reagents and Instruments

Nicotine (analytical pure) was provided by Yunnan Tobacco Industry Co., Ltd. All chemicals were analytical grade and can be directly used without further purification. 0.1 m phosphate‐buffered saline (PBS) was prepared by mixing 0.1 m NaH_2_PO_4_ with 0.1 M Na_2_HPO_4_, and the pH was adjusted using H_3_PO_4_ or NaOH. Deionized water was used in all experiments.

The electrochemical measurements were performed on a PalmSens4 electrochemical workstation (Red Matrix China LTD) with a smartphone (OPPO) and SPAS (sinjeen200, Changsha Sunjun Electronic Technology Co., Ltd). All potential was measured and reported relative to the Ag/AgCl (0.222 V) as the reference electrode on the SPAS platform. The sensor was characterized by ultraviolet–visible spectrometer (Shanghai Jinghua, China), Czech TESCAN MIRA LMS scanning electron microscope (SEM), United States Thermo Scientific K‐Alpha X‐ray Photoelectron Spectroscopy (XPS), and CA100 Contact Angle Measurement (Guangdong, China).

### 4.2 Calculation Details

All molecular modeling and orbital analyses were carried out with GaussView 6.0, while quantum chemical calculations were performed using Gaussian 09 and Deep Chemistry. Geometry optimizations and energy evaluations including electrostatic potential (ESP), HOMO–LUMO gap, and Fukui function analyses, were conducted at the B3LYP/6‐311+G(d) level of theory. The same functional and basis set were employed for optimizing structures visualized via localized orbital locator‐π (LOL‐π) isosurfaces. For adsorption energy studies and weak interaction systems, geometry optimizations were performed at the B3LYP/6‐31G(d) level, with single‐point energy calculations carried out at the B3LYP/DEF2‐TZVP level. Transition state structures were optimized using the B3LYP/6‐31G(d,p) level, and their energies were refined at the PWPB95/DEF2‐TZVPP level. In all cases, dispersion corrections were incorporated using Grimme's D3 method with Becke–Johnson damping (GD3BJ).

Ab initio calculations were performed using the VASP code [66]. Geometry optimization was conducted using the projector‐augmented‐wave (PAW) pseudopotentials with a plane–wave basis set. The exchange–correlation interactions were treated within the generalized gradient approximation (GGA) using the Perdew–Burke–Ernzerhof (PBE) functional, while van der Waals (VdW) interactions were incorporated via Grimme's D2 dispersion correction [67]. A plane–wave energy cutoff of 400 eV was used, and the energy convergence criterion was set to 10^−^⁵ eV. The first Brillouin zone was sampled with a 3×3×1 Γ‐centered k‐point mesh. During geometry optimization, both atomic positions and lattice parameters were fully relaxed until the residual forces fell below 0.01 eV/Å. After obtaining the ground‐state structures at 0 K, a 5 ps ab initio molecular dynamics (AIMD) simulation was performed in the NVT ensemble with a time step of 1 fs. The temperature was maintained at 300 K using the Nosé–Hoover thermostat.

Using multiwfn to optimize the electrostatic potential structure [68]. All input structure coordinates can be found in Section S2.

### 4.3 Multi‐Scenario NIC Detection Based on SPAS Portable Sensor

**SCHEME 1:**
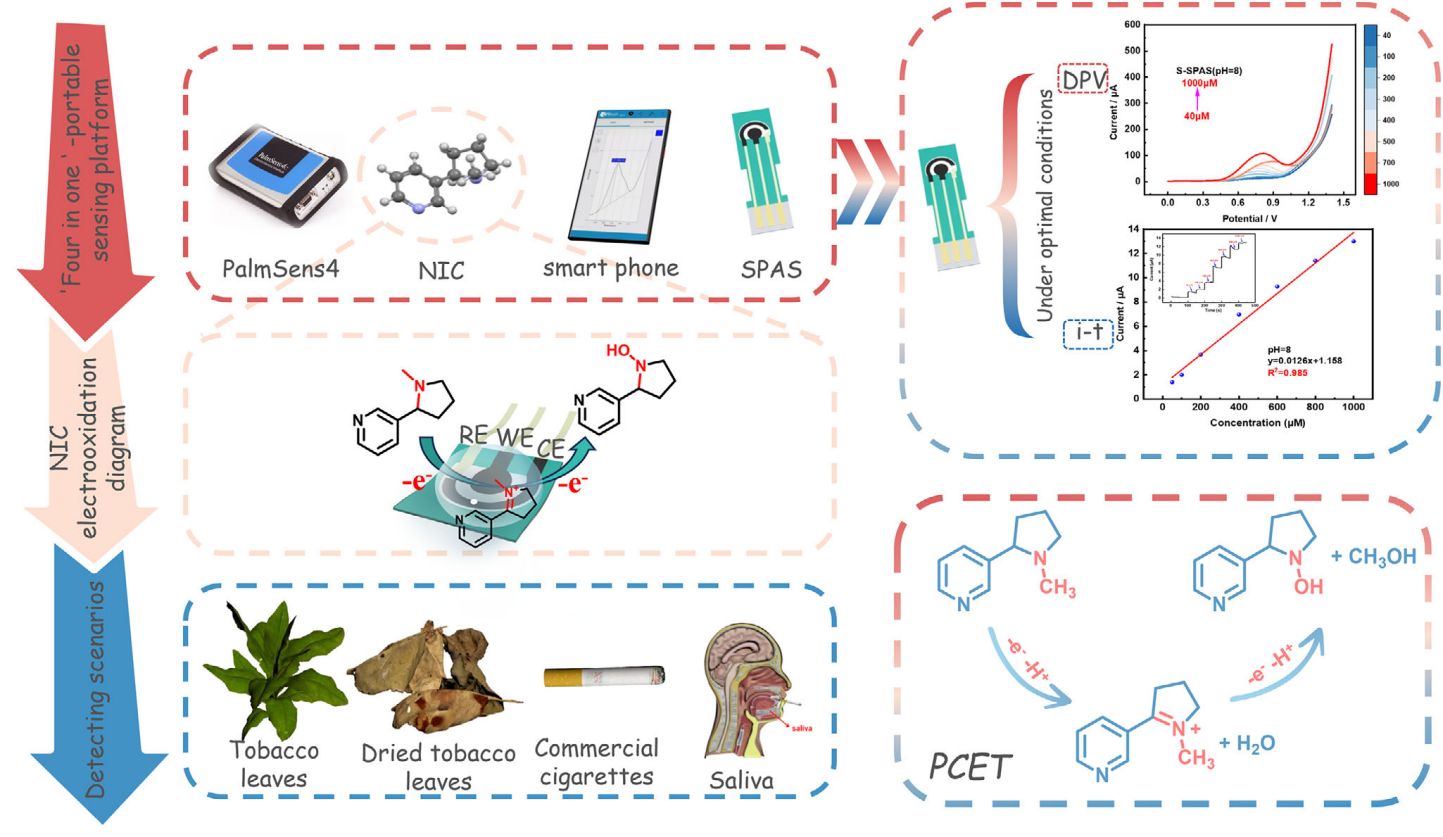
Structure diagram of the NIC portable electrochemical sensor.

A portable electrochemical sensor was constructed by connecting the portable electrochemical workstation and smartphone through Bluetooth. All electrochemical experiments were carried out in 0.1 m PBS. NIC was detected by DPV and I–T at SPAS. DPV setting parameters: scanning potential 0∼1.3 V, potential increment 0.01 V, and scanning rate 0.1 V/s. I–T setting parameters: initial potential 0.85 V, running time 450 s, equilibration time 2 s, sample interval 0.1 s. The portable sensor is used for multi‐scenario electrochemical detection of NIC (tobacco leaves, dried tobacco leaves, cigarettes, and saliva of smokers). The accuracy of the results obtained through the standard addition method. In summary, the sensing platform is shown in Scheme 1.

### 4.4 Treatment of NIC Sample

In the ultrasonic extraction section, the ultrasonic frequency is 40 KHz, and extraction is carried out at room temperature (25 ± 2°C). The instrument operates at its maximum power setting, and the sample beaker is placed in the center of the cleaning tank. The water level in the ultrasonic cleaning tank is kept constant to ensure effective energy transfer. A volume of 200 mL of ethanol is optimal for the size of the cleaning tank to ensure effective cavitation. For different samples, including fresh tobacco leaves, dried tobacco leaves, and cigarette samples, appropriate amounts were weighed and completely immersed in ethanol solution for 5‐min ultrasonic extraction. After extraction, accurately measure 2 mL of the extraction solution using a precision pipette and transfer it to a container containing an appropriate amount of PBS buffer (pH 8.0). Mix well and prepare for subsequent testing. For saliva samples from smokers, a certain volume of saliva needs to be collected first, centrifuged at 6000 rpm for 10 min, and the supernatant is directly mixed with PBS buffer (pH 8.0) in a certain proportion without ultrasound treatment before entering the detection stage.

In the in situ detection of live tobacco leaves, select tobacco leaves from the upper, middle, and lower positions of a tobacco plant, and randomly detect three different positions of the same tobacco leaf. When performing local minimally invasive treatment, use a sterile punch with a radius of 1 mm to create a micro injury area on the leaf surface, paying attention to controlling the depth to avoid tissue penetration. Subsequently, 100 µL of PBS buffer (pH 8.0) was accurately transferred using a micropipette and gently dropped onto the minimally invasive surface to ensure complete coverage of the wound area. Use cleaned glass sheets to firmly clamp live tobacco leaves with the SPAS sensor platform, and directly detect the released NIC through the sensor, providing accurate NIC concentration information immediately [69].

## Author Contributions

Zhaohong Su, Qian Liu, Lihui Ou, and Shiyu Hu are the corresponding authors of this article and contributed to its development of this article. Yi Peng, Qinyi Cao, Qianyu Shen, Yuhang Zhang, and Hongdou Yi contributed to the development of the experimental part. Yi Peng, Qinyi Cao, and Lihui Ou made contributions to the theoretical calculation part of this article. Qiang Li provided actual samples for this article. Zhaohong Su, Qian Liu, and Shiyu Hu participated in the correction of the manuscript.

## Conflicts of Interest

The authors declare no conflicts of interest.

## Supporting information




**Supporting File**: advs73528‐sup‐0001‐SuppMat.docx.

## Data Availability

Research data are not shared.
