# Supplementary material for: Decoding Proton‐Coupled Electron Transfer Mechanism of Nicotine for Multi‐Scenario Portable Electrochemical Sensing
Source: Adv Sci (Weinh). 2025 Dec 22;13(14):e21198. doi: 10.1002/advs.202521198 (PMC12970285; doi:10.1002/advs.202521198)
Supplement: Supplementary file 1 — Supporting File: advs73528‐sup‐0001‐SuppMat.docx. [file ADVS-13-e21198-s001.docx]

**Supporting Information**

**Decoding proton-coupled electron transfer mechanisms of nicotine for multi-scenario portable electrochemical sensing**

*Yi Peng^1^, Qinyi Cao ^1^, Qianyu Shen, Yuhang Zhang, Shiyu Hu*, Hongdou Yi,* *Qian Liu *, Lihui Ou*, Qiang Li, Zhaohong Su**

Y. Peng, Q. Cao, Q. Shen, Y. Zhang, S. Hu, H. Yi, Q. Li, Z. Su

College of Chemistry and Materials Science, College of Agronomy, Hunan Agricultural University； Rapeseed Variety Creation Center Team, Yuelushan Laboratory

Changsha 410128, China

E-mail address: zhaohongsu@hunau.edu.cn (Zhaohong Su); hushiyu@stu.hunau.edu.cn (Shiyu Hu)

Q. Liu

State Key Laboratory of Environmental Chemistry and Ecotoxicology, Research Center for Eco-Environmental Sciences, Chinese Academy of Sciences,

Beijing 100085, China

E-mail address: qianliu@rcees.ac.cn (Qina Liu)

L. Ou

College of Chemistry and Materials Engineering, Hunan University of Arts and Science

Changde 415000, China

[lihuiou@huas.edu.cn](mailto:lihuiou@huas.edu.cn) (Lihui Ou)

*Corresponding author. Tel.: +86 731 8461807; fax: +86 731 84618071

^1^These authors contributed equally to this work

**Section 1**

^
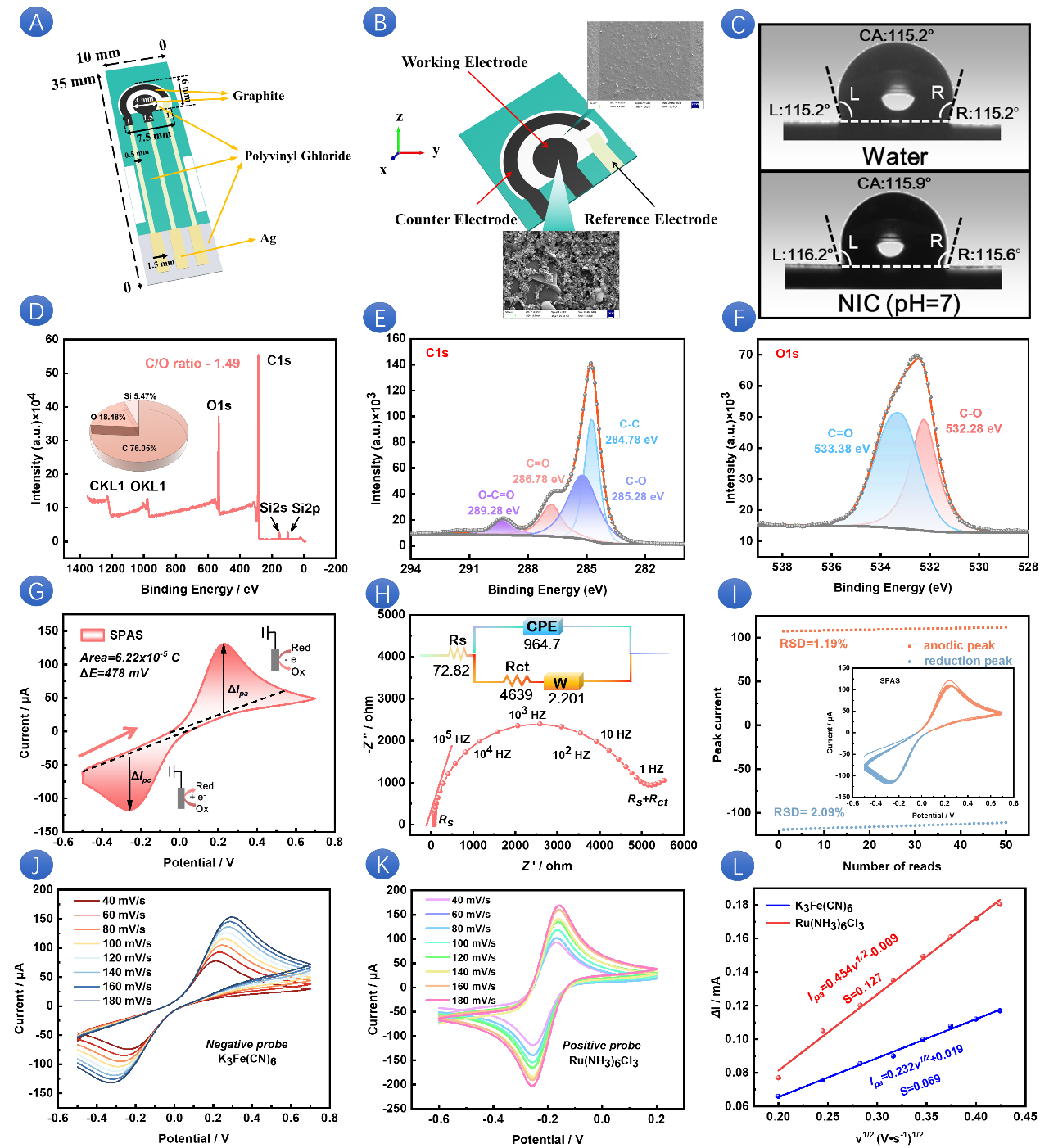
^

**Figure S1.** Size and composition material model of SPAS (A). Working area model of SPAS and SEM images of Working Electrode and electrode blank area (B). Comparison of Contact Angle between water and NIC (pH=7) on SPAS (C). XPS full spectrum and element content of SPAS (D). XPS peak fitting of C (E) and O (F). CV (G) and EIS (H) of SPAS in 5.0 mM [Fe(CN)_6_]^3−/4−^+ 0.1 M KCl solution, respectively. Scanning rate: 100 mV/s, EIS parameters: 100 kHz to 1 mHz, 5 mV rms, open circuit potential. Illustration is a circuit fitting diagram. RSD of the redox peaks from 50 cycles of CV in 0.1 M KCl containing 5.0 mM [Fe(CN)_6_]^3−/4−^, the inset showing the CV curves for 50 cycles (I). CV of SPAS in 5.0 mM [Fe(CN)_6_]^3-/4-^ + 0.1 M KCl and 5.0 mM [Ru(NH_3_)_6_]^2+/3+^ + 0.1 M KCl, scan rate of 40~180 mV/s (J-K). Linear relationship between *I*_pa_ and *v*^1/2^ (L).







(Enlarged SEM images of Working Electrode (Right) and electrode blank area (Left) in Figure S1B)


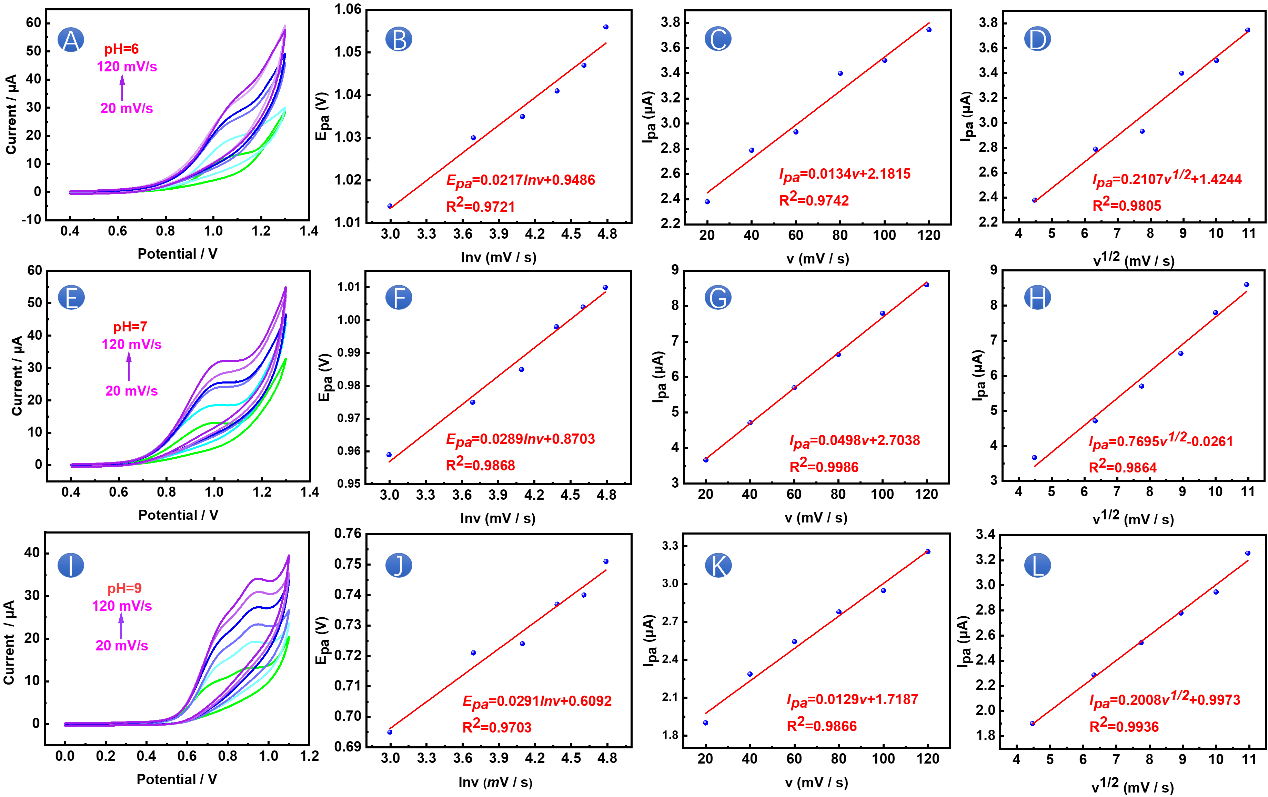


**Figure S2.** CVs of SPAS in PBS with different pH containing 500 μM NIC. Linear correlation between *E* and *lnv* (B, F, J), *I*_pa_ and *v* (C, G, K), *I*_pa_ and *v*^1/2^ (D, H, L).


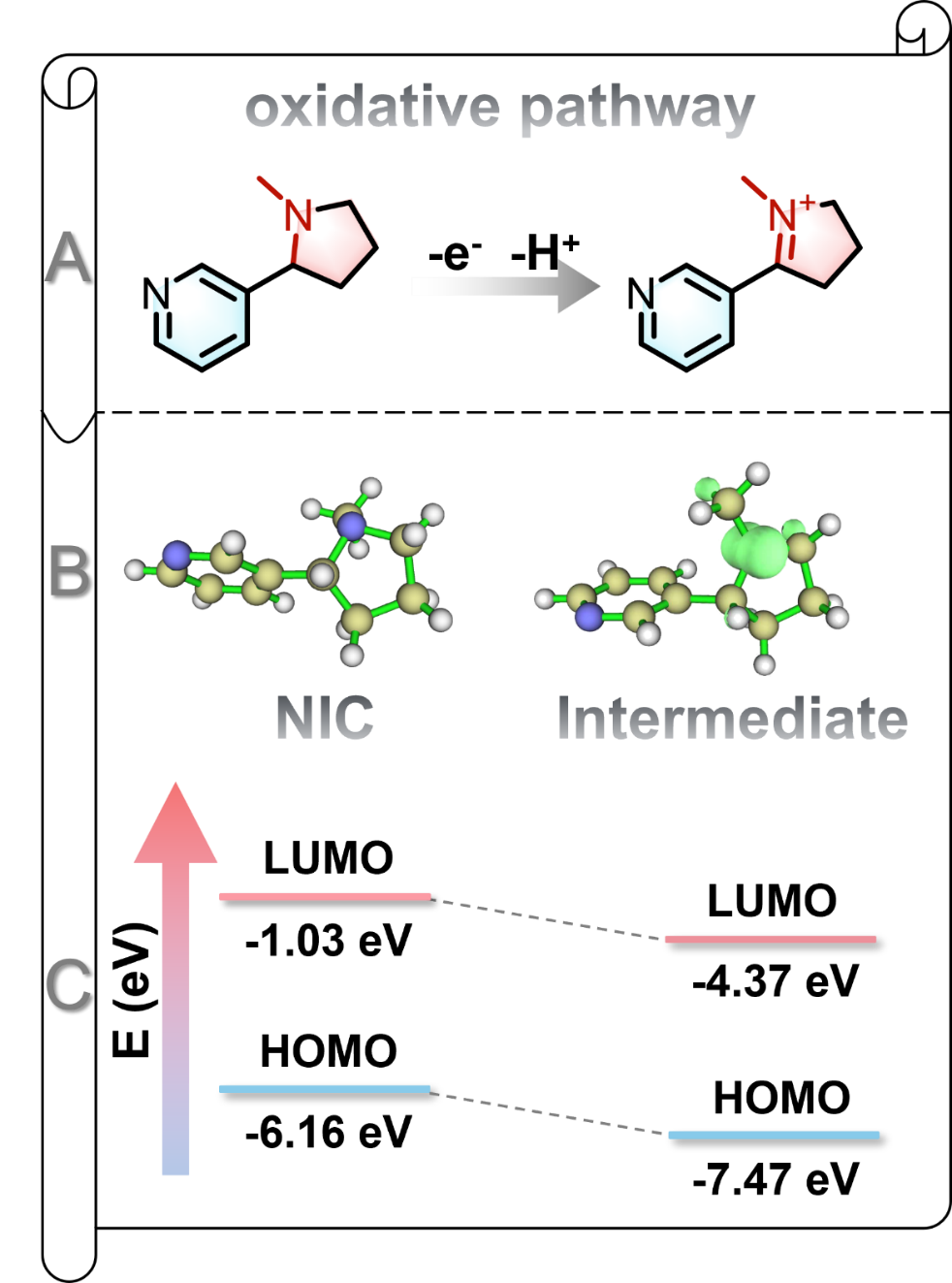


**Figure S3.** Intermediate formation pathway (A). Spin isodensity surfaces of NIC and Intermediate (B). HOMO-LUMO energy of NIC and Intermediates (C).


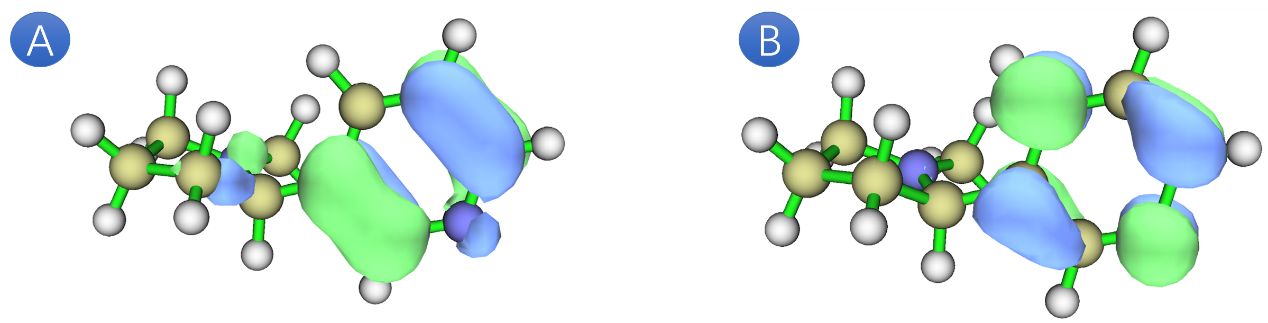


**Figure S4.** HOMO(A)-LUMO(B) of Intermediates


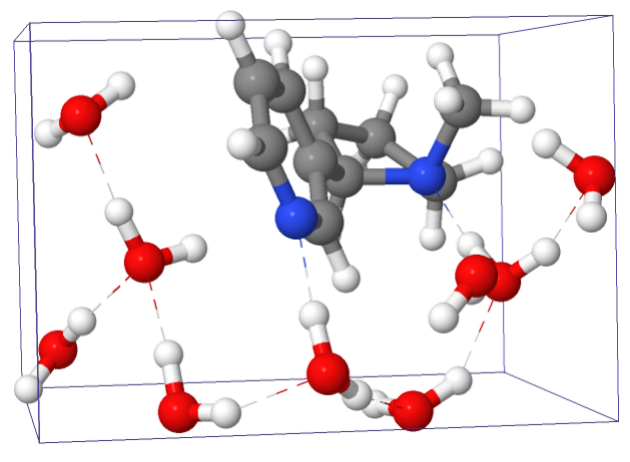


**Figure S5.** Schematic diagram of interaction between NIC and H_2_O.


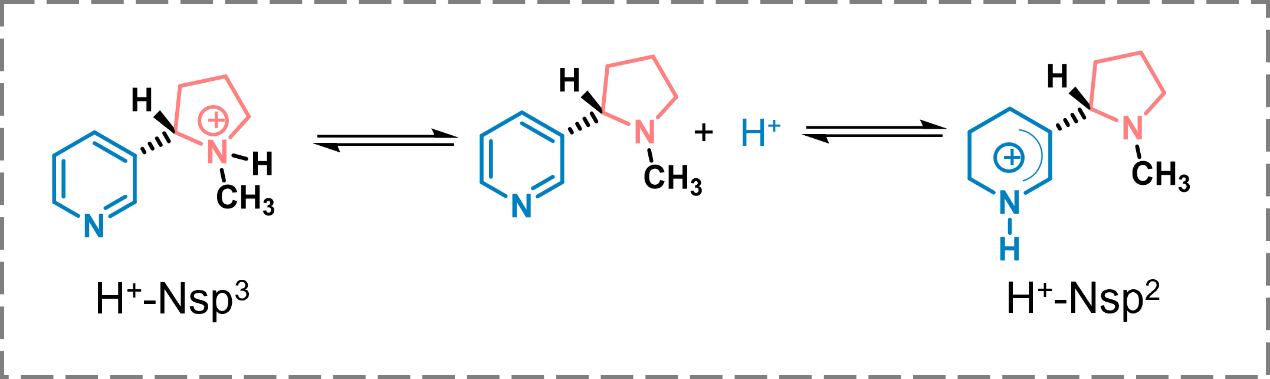


**Figure S6.** Possible location of the first protonation of NIC.


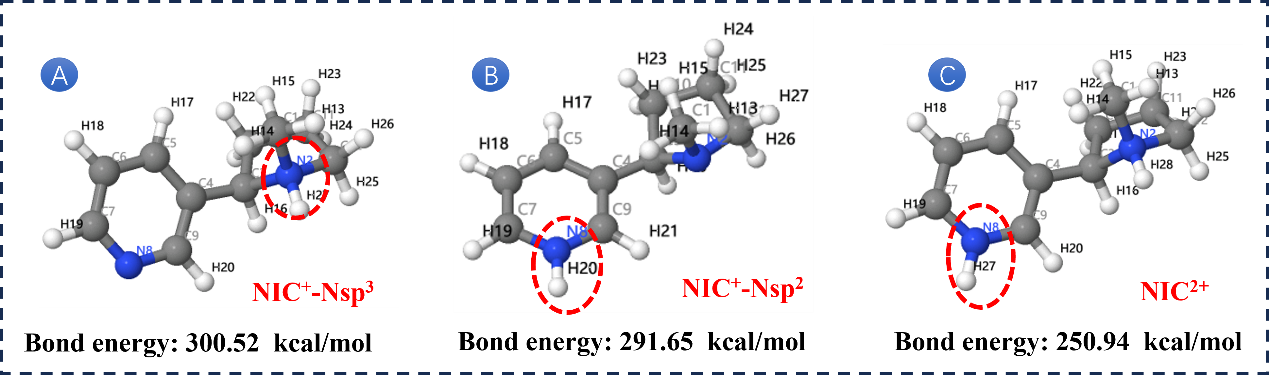


**Figure S7.** Bond energy analysis of NIC protonation at different positions.


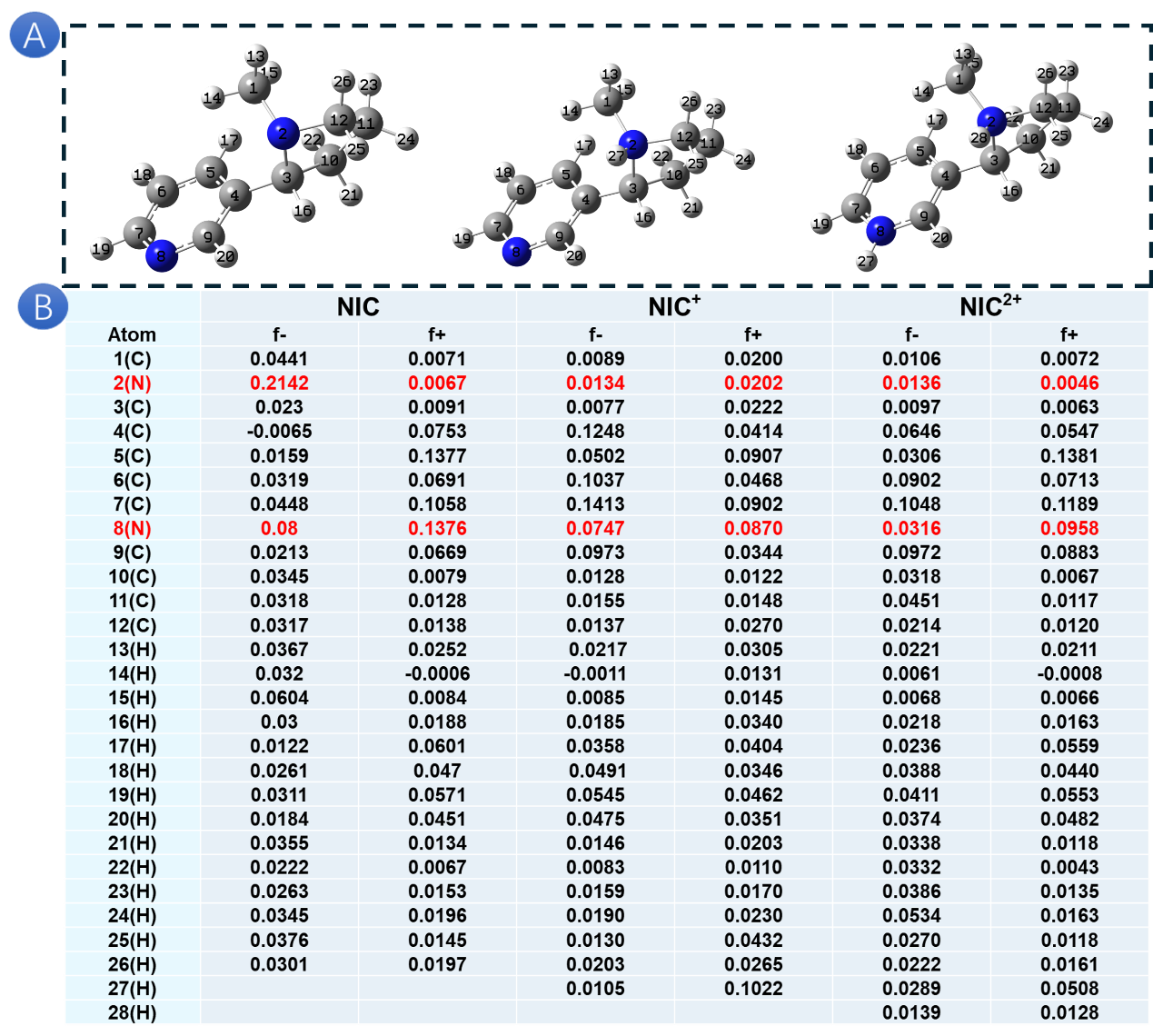


**Figure S8.** Structure (A) and Fukui index (B) of NIC, NIC^+^, NIC^2+^.


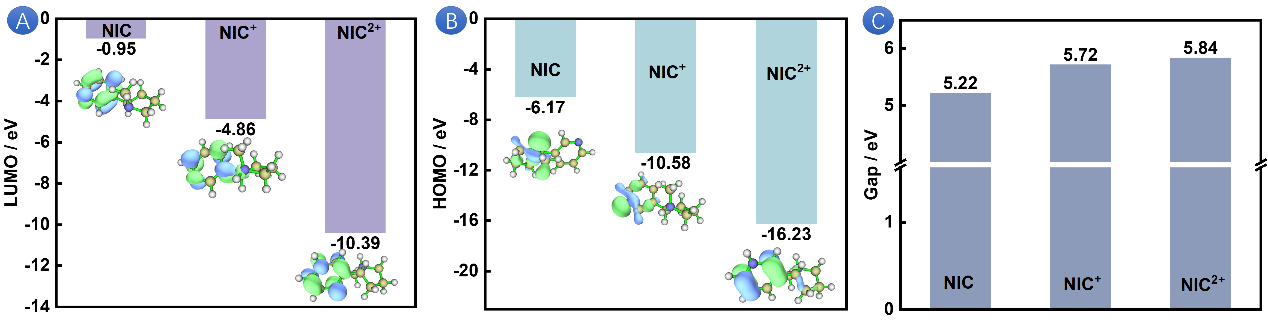


**Figure S9.** HOMO-LUMO gap calculations of NIC, single-proton NIC and double-proton NIC in vacuum.


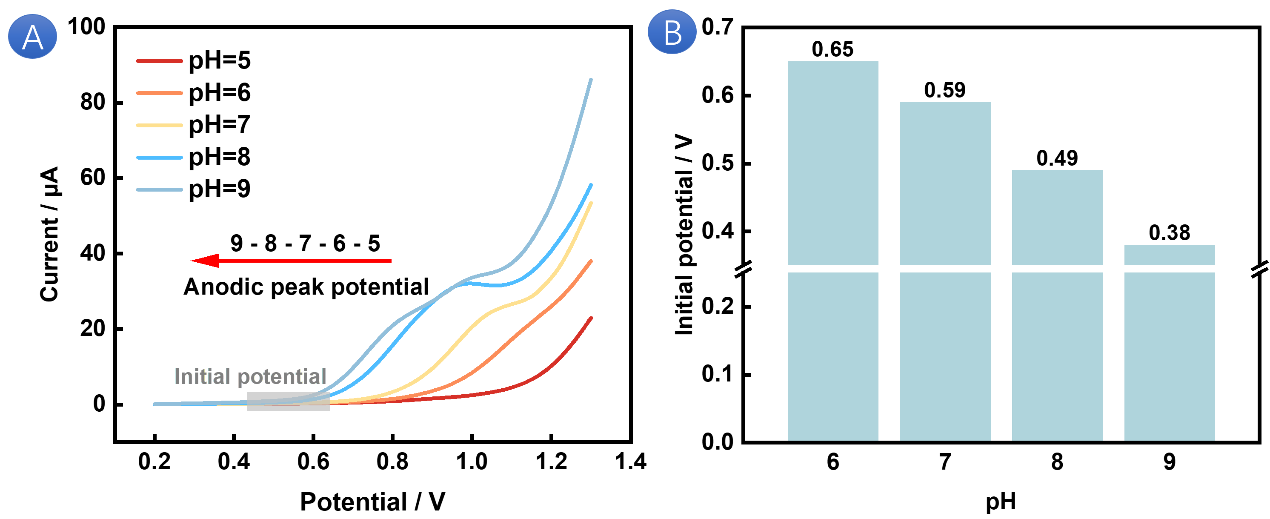


**Figure S10.** LSV curve of SPAS in PBS containing 500 μM NIC with different pH (5~9) (A). Oxidation onset potential of at different pH in LSV (B).


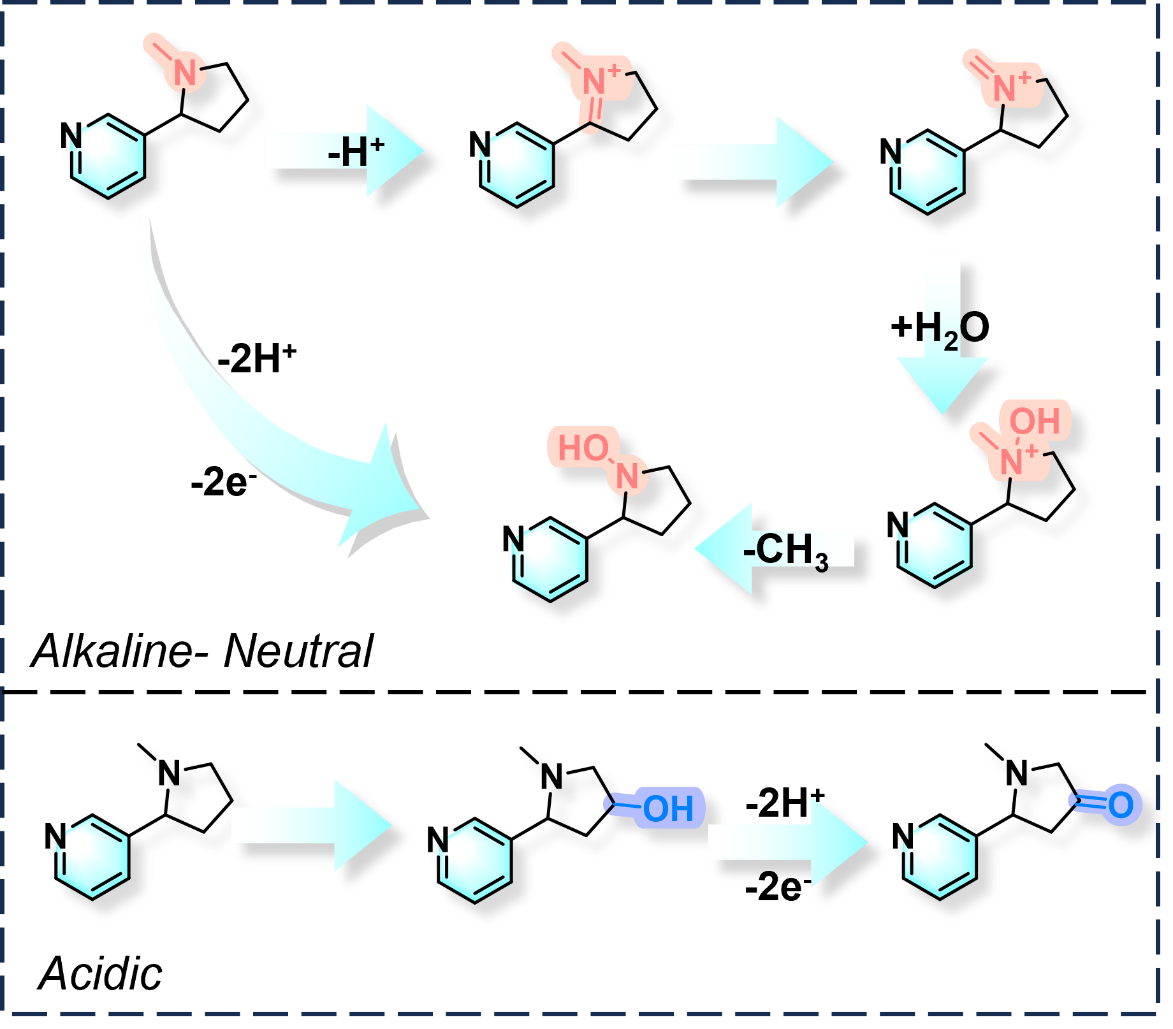


**Figure S11.** Possible oxidation mechanism of NIC at different pH.


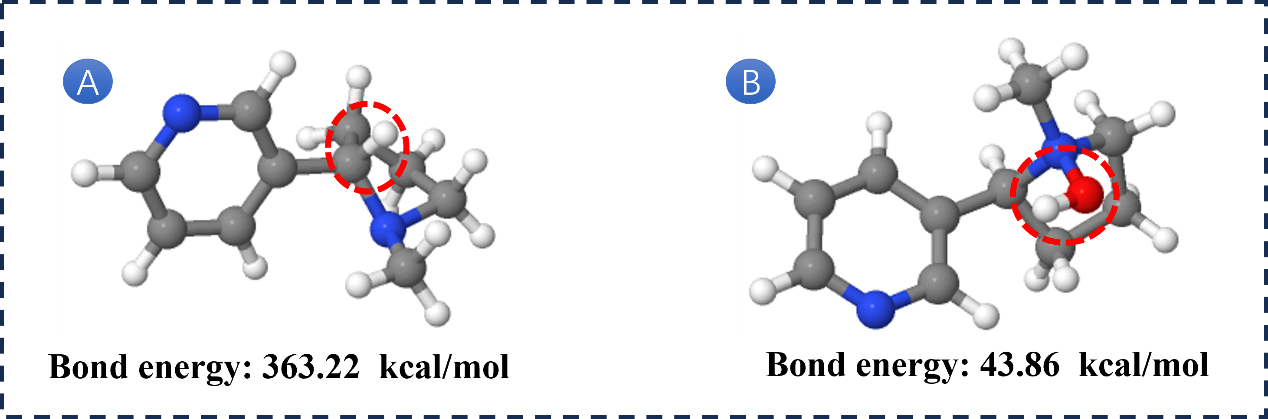


**Figure S12.** Bond energy analysis of C-H (A) and C-OH (B).


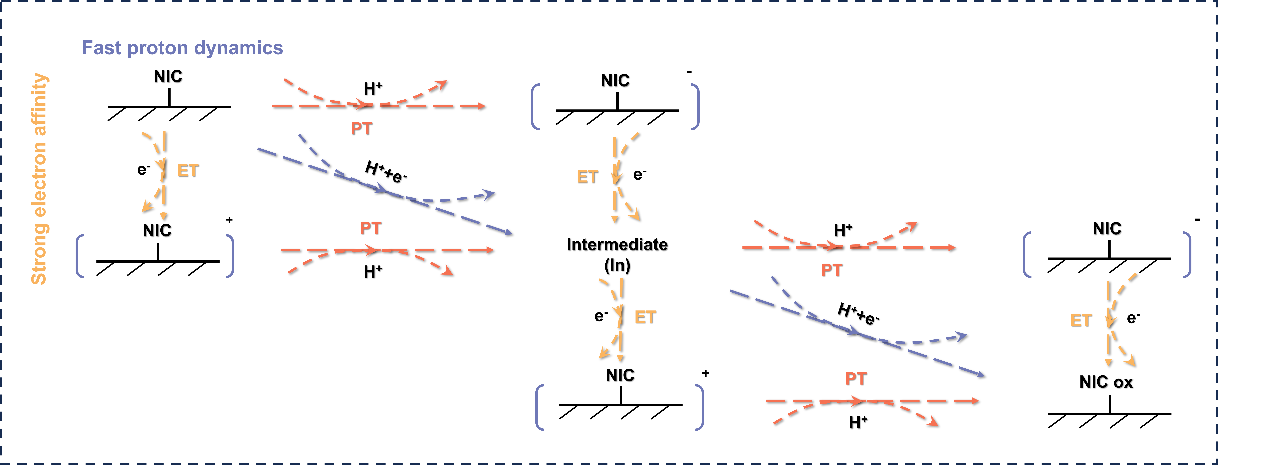


**Figure S13.** PCET transfer steps for NIC electrooxidation process.


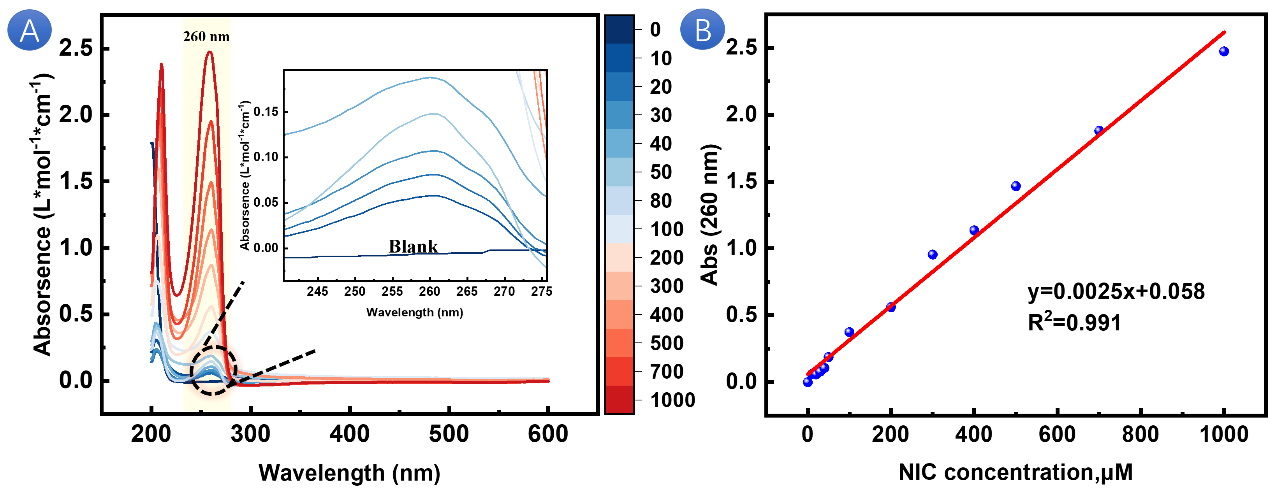


**Figure S14.** UV-vis spectrum of different NIC concentration (A). Linear relationship between Abs and NIC concentration (B). The illustration shows the UV-vis spectrum of NIC concentration (0-50 μM).


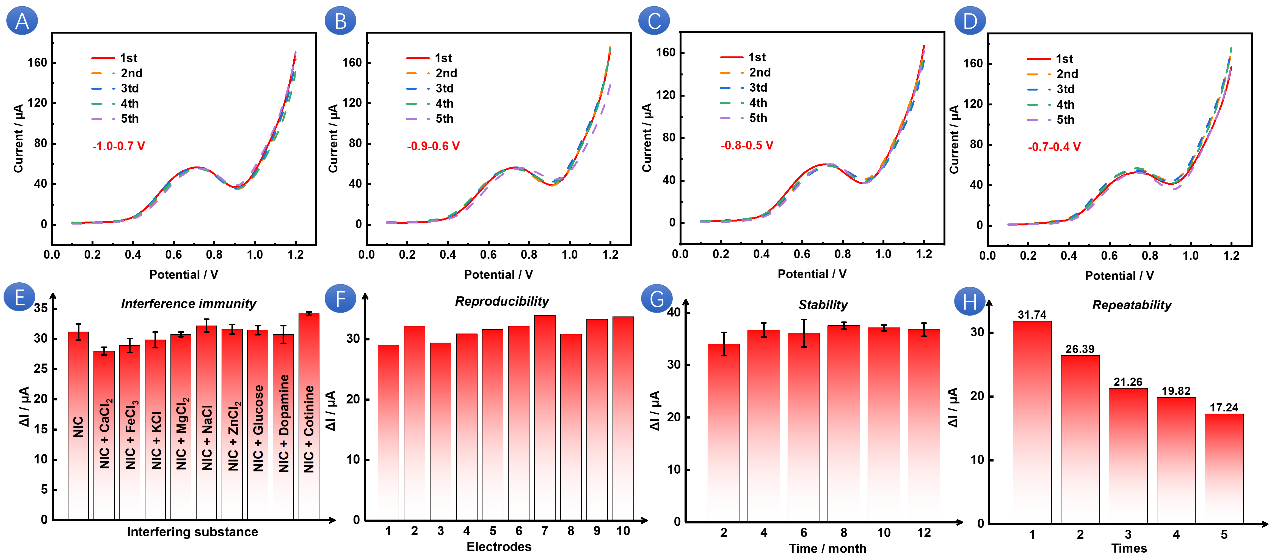


**Figure S15.** DPV of SPAS treated with different CV scanning potential ranges in 0.1 M PBS (pH=8) containing 500 μM NIC (A-D). Comparison of DPV peak current of SPAS in 0.1M PBS (pH=8) containing 500 μM NIC and different interfering substances (E). Reproducibility (F), stability (G) and repeatability (H) of SPAS in 0.1 M PBS (pH = 8) containing 500 μM NIC.


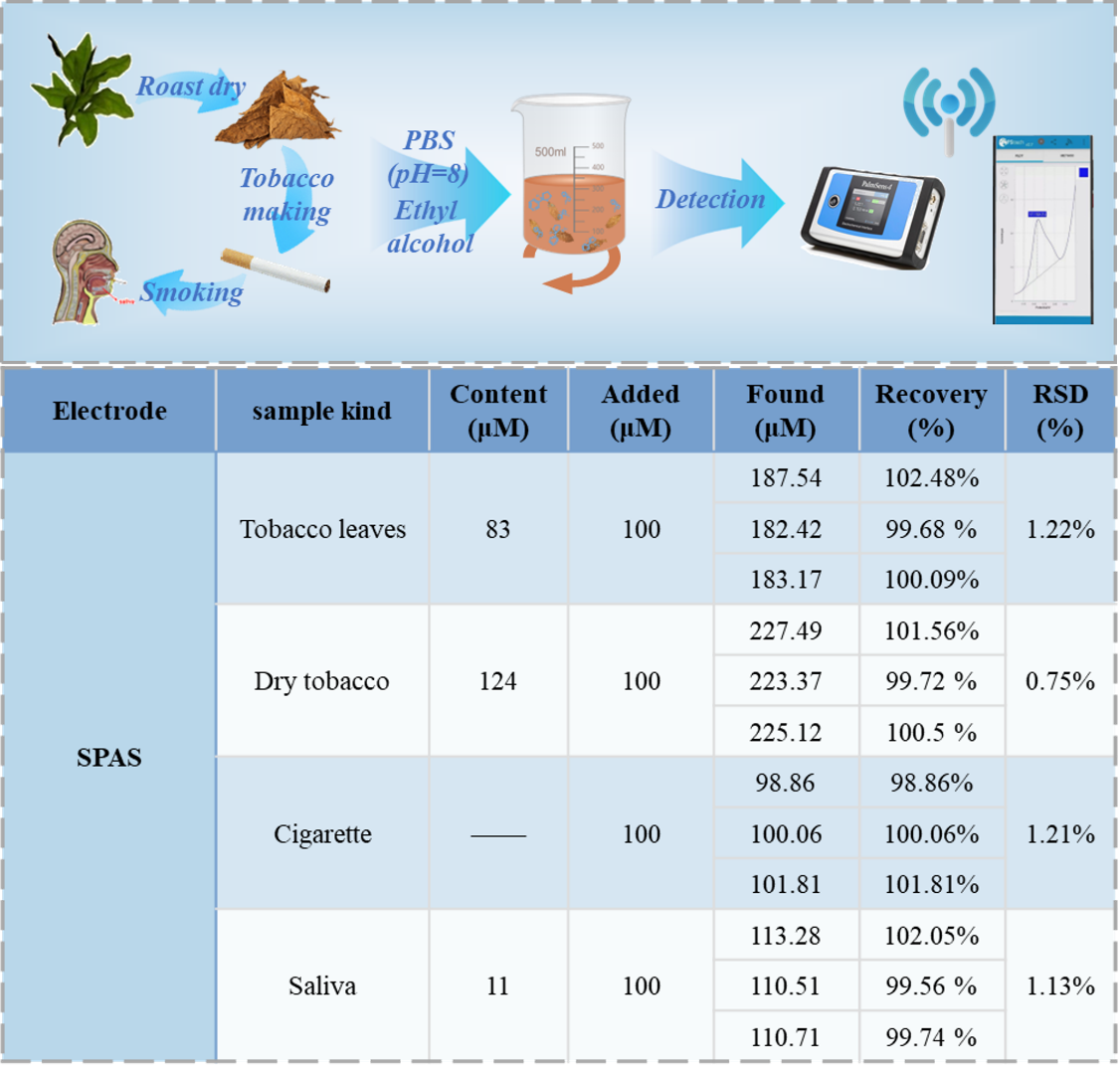


**Figure S16.** NIC static detection in four different objects based on SPAS portable sensor (n=3).


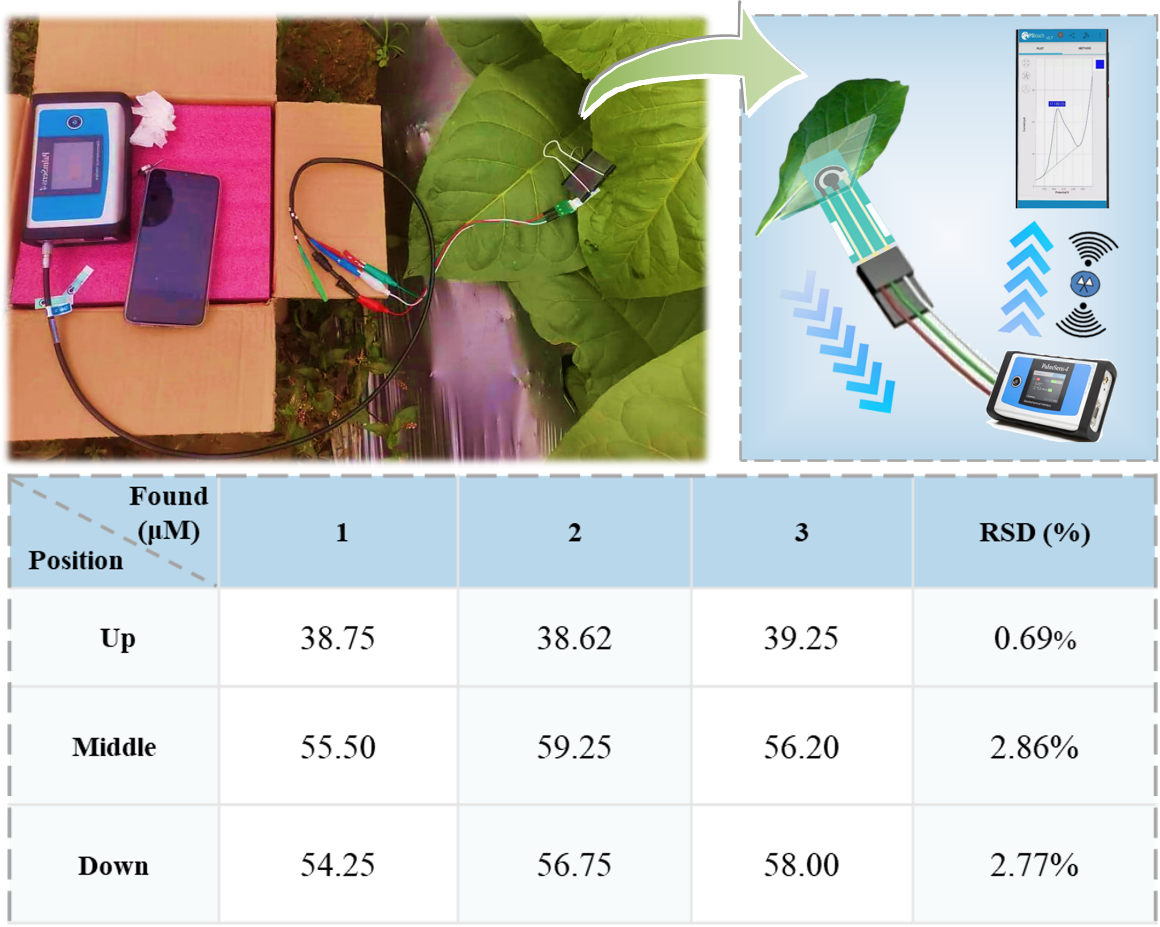


**Figure S17.** Dynamic detection of NIC in tobacco leaves based on SPAS portable sensor (n=3).

**Table S1.** Morphological distribution of NIC in PBS with different pH 5-9.

| pH | Free NIC (%) | Single-proton NIC (%) | Double-proton NIC (%) |
| --- | --- | --- | --- |
| 5 | 0.03 | 99.47 | 0.50 |
| 6 | 0.26 | 99.68 | 0.06 |
| 7 | 2.58 | 97.41 | 0.01 |
| 8 | 20.93 | 79.07 | 0.00 |
| 9 | 72.58 | 27.42 | 0.00 |

**Table S2.** Thermodynamic parameters of each substance in the process of NIC protonation.

| **Calculation material** | **Enthalpy change**  **(H kcal/mol)** | **Gibbs free energy**  **(G kcal/mol)** | **Single point energy**  **(E kcal/mol)** | **Dipole moment**  **(C·m)** |
| --- | --- | --- | --- | --- |
| **NIC (Water)** | -312850.2683 | -312880.0374 | -312999.7704 | 3.37969 |
| **NIC^+^ (Water)** | -313133.1755 | -313163.0516 | -313292.8693 | 9.70653 |
| **NIC^2+^ (Water)** | -313406.6240 | -313437.8489 | -313574.8273 | 4.55210 |
| **H_2_O** | -47941.1037 | -47954.5552 | -47956.7491 | 2.51142 |
| **H_3_O^+^** | -48198.2774 | -48212.6808 | -48222.4925 | 2.18253 |
| **NIC (Gas)** | -312844.1424 | -312873.9156 | -312993.8314 | 2.53199 |
| **NIC^+^ (Gas)** | -313072.2703 | -313102.5628 | -313231.6008 | 7.06372 |
| **NIC^2+^ (Gas)** | -313223.5786 | -313254.3125 | -313391.6893 | 3.43316 |

**Table S3.** Comparison of two detection methods based on SPAS.

| **Electrode** | **Method** | **pH** | **Detection range (μM)** | **Linear equation** | **Sensitivity**  **(μA·μM⁻¹·cm⁻²)** | **LOD (μM)** | **LOQ (μM)** |
| --- | --- | --- | --- | --- | --- | --- | --- |
| SPAS | DPV | 6 | 300-1000 | y=0.033x-10.29 | 2.62×10^-3^ | 135.3 | 409.9 |
|  |  | 7 | 50-1000 | y=0.050x-1.03 | 3.98×10^-3^ | 25.08 | 76.10 |
|  |  | 8 | 40-1000 | y=0.080x-2.34 | 6.38×10^-3^ | 17.01 | 51.54 |
|  | I-T | 5 | 50-1000 | y=0.0016x+0.21 | 1.29×10^-4^ | 49.50 | 150.2 |
|  |  | 6  7 |  | y=0.0026x+0.16  y=0.0090x+0.75 | 2.04×10^-4^  7.18×10^-4^ | 30.46  22.73 | 92.31 |
|  |  |  |  |  |  |  | 68.89 |
|  |  | 8 |  | y=0.013 x+1.16 | 1.00×10^-3^ | 16.50 | 49.95 |
|  |  | 9 |  | y=0.0094x+1.30 | 7.51×10^-4^ | 22.47 | 68.09 |

**Table S4.** Comparison of different electrochemical sensing properties for NIC detection.

| Material | Multi-scenario application | | Electrodes | Detection Range/µmol/L | | | Detection  Limit/µmol/L | | Detection  Method | Supporting  electrolyte |
| --- | --- | --- | --- | --- | --- | --- | --- | --- | --- | --- |
| Mo_2_C/C^[1]^ | | No | GCE | 0.2-300 | | 0.17 | | CV | | 0.1 M PBS  (pH 7.4) |
| CS/MWCNT-COOH^[2]^ | | No | GC | | 0.1-100 | 30 | | DPV | | 0.1 M PBS  (pH 7.4) |
| GO/Nq^[3]^ | | No | GCE | | 6.5-245 | 12.7 | | DPV | | 0.1M Na_2_SO_4_  (pH=8.0) |
| BN/graphene^[4]^ | | No | GCE | | 1-1000 | 0.42 | | I-T | | 0.1M PBS  (pH=7.0) |
| ERCG^[5]^ | | No | GCE | | 2-5,2-60 | 0.1 | | CV | | 0.1M PBS  (pH=7.0) |
| BITSH-1^[6]^ | | No | GCE | | 0-50 | 0.25 | | SWV | | 0.1M PBS  (pH=7.04) |
| PDA-RGO/Au^[7]^ | | No | GCE | | 0.05-500 | 0.015 | | I-T | | 0.1M BR  (pH=7.5) |
| MX/Gr^[8]^ | | No | GCE | | 0.03-0.6  1-55 | 0.28×10^-3^ | | DPV | | 0.1M PBS  (pH=7.4) |
| Ag-NPs^[9]^ | | No | GCE | | 2.5-105 | 0.135 | | I-T | | 0.1M PBS  (pH=7.4) |
| Graphite  (This work) | Yes | | SPAS | | 40-1000  50-1000 | 17.01 | | DPV | | 0.1M PBS  (pH=8.0) |
|  |  |  |  |  |  | 16.50 | | I-T | |  |

*CS/MWCNT-COOH: Carbon nanotube hybrid nanofibers; GO/Nq: 1,2-naphthoquinone-4-sulfonic acid conjugated graphene oxide nanoparticles; BN/graphene: Hexagonal boron nitride doped graphene; ERCG: Electroreduced carboxylated graphene; BITSH-1: Metal-organic framework; PDA-RGO/Au: Polydopamine functionalized reduced graphene oxide-gold nanoparticle; MX/Gr: Two-dimensional nitride/graphene; Ag-NPs: silver nanoparticles; Mo_2_C/C: Preparation of Mo_2_C nanoparticles from carbon precursors and their dispersion on carbon nanosheets.

**References** (Only for Supporting Information)

[1] Y. Du, M. M. Ahmed, T. Xing, S. Chen, J. Du, J.Chem.Phys. **2025**, 162, 014706.

[2] A. Mirani, L. Maleknia, A. Amirabadi, *Nanotechnology.* **2020**, 31, 435504.

[3] M. Abd-Elsabour, H. M. Alsoghier, A. G. Alhamzani, M. M. Abou-Krisha, T. A. Yousef, H. F. Assaf, *Nanomaterials*. **2022**. 1214 2354

[4] R. Jerome, A. K. Sundramoorthy, *Anal. Chim. Acta.* **2020**, 1132, 110.

[5] H. Xiao, L. Sun, H. Yan, W. Wang, J. Liu, Q. Yan, L. Chao, C. Chen, Q. Xie, J. Wen, D. Yin, *Anal. Methods.* **2015**, 7, 1147.

[6] T. Leelasree, S. Goel, H. Aggarwal, *ACS Appl. Nano Mater.* **2022**, 5, 16753.

[7] Y. Jing, X. Yuan, Q. Yuan, K. He, Y. Liu, P. Lu, H. Li, B. Li, H. Zhan, G. Li, *Scientific Reports* **2016**, 6, 29230.

[8] J. Rajendran, A. K. Sundramoorthy, D. Ganapathy, R. Atchudan, M. A. Habila, D. Nallaswamy, *J. Hazard. Mater.* **2022**, 440, 129705.

[9] G. Sridharan, K. L. Babu, D. Ganapathy, R. Atchudan, S. Arya, A. K. Sundramoorthy, *Crystals*. **2023**. 134, 589.

| **Section2**  Protonated | | | |
| --- | --- | --- | --- |
| NIC |  |  |  |
| C | 1.319825 | 0.840042 | 1.608405 |
| N | 1.502533 | -0.44984 | 0.926942 |
| C | 0.7403 | -0.53194 | -0.3574 |
| C | -0.73069 | -0.23075 | -0.22772 |
| C | -1.27323 | 1.05399 | -0.29766 |
| C | -2.64589 | 1.223411 | -0.14783 |
| C | -3.44055 | 0.103094 | 0.067102 |
| N | -2.9478 | -1.14228 | 0.1336 |
| C | -1.62388 | -1.28349 | -0.01367 |
| C | 1.536107 | 0.311908 | -1.38198 |
| C | 2.995202 | 0.230318 | -0.87639 |
| C | 2.893676 | -0.57683 | 0.440328 |
| H | 1.978756 | 0.871783 | 2.477168 |
| H | 0.294833 | 0.935246 | 1.966161 |
| H | 1.544049 | 1.715232 | 0.984023 |
| H | 0.825485 | -1.57779 | -0.66561 |
| H | -0.63861 | 1.915591 | -0.46513 |
| H | -3.09574 | 2.208271 | -0.19711 |
| H | -4.51428 | 0.205058 | 0.187642 |
| H | -1.24383 | -2.29991 | 0.037985 |
| H | 1.410246 | -0.09247 | -2.38807 |
| H | 1.190638 | 1.345952 | -1.40381 |
| H | 3.401916 | 1.227644 | -0.69772 |
| H | 3.655609 | -0.26721 | -1.58926 |
| H | 3.089608 | -1.63388 | 0.24204 |
| H | 3.593044 | -0.2472 | 1.208774 |
|  |  |  |  |
|  |  |  |  |
| NIC^1+^ |  |  |  |
| C | 1.255033 | -0.08426 | 1.86061 |
| N | 1.495753 | -0.78295 | 0.565839 |
| C | 0.710643 | -0.22169 | -0.63082 |
| C | -0.75558 | -0.0509 | -0.34683 |
| C | -1.31073 | 1.106771 | 0.200643 |
| C | -2.6804 | 1.149528 | 0.434711 |
| C | -3.45027 | 0.036185 | 0.11634 |
| N | -2.93985 | -1.08473 | -0.41429 |
| C | -1.62088 | -1.1088 | -0.63556 |
| C | 1.535779 | 1.000339 | -1.03899 |
| C | 2.996613 | 0.653169 | -0.66223 |
| C | 2.927875 | -0.67625 | 0.100843 |
| H | 1.905848 | -0.53328 | 2.607219 |
| H | 0.215829 | -0.21539 | 2.148425 |
| H | 1.486819 | 0.972303 | 1.752342 |
| H | 0.83046 | -1.00055 | -1.38451 |
| H | -0.69358 | 1.961897 | 0.446669 |
| H | -3.14669 | 2.031119 | 0.857986 |
| H | -4.52116 | 0.04247 | 0.290407 |
| H | -1.22255 | -2.02053 | -1.07142 |
| H | 1.406296 | 1.182438 | -2.10507 |
| H | 1.208276 | 1.89397 | -0.50991 |
| H | 3.430536 | 1.436414 | -0.04133 |
| H | 3.62953 | 0.537853 | -1.54135 |
| H | 3.101272 | -1.53088 | -0.5506 |
| H | 3.585501 | -0.74389 | 0.963802 |
| H | 1.243788 | -1.76479 | 0.684214 |
|  |  |  |  |
|  |  |  |  |
| NIC^2+^ |  |  |  |
| C | 1.312768 | 0.66976 | 1.741641 |
| N | 1.540923 | -0.49383 | 0.834863 |
| C | 0.74943 | -0.46849 | -0.47662 |
| C | -0.71523 | -0.18219 | -0.27895 |
| C | -1.25208 | 1.106645 | -0.19158 |
| C | -2.61909 | 1.291476 | -0.00666 |
| C | -3.44274 | 0.1889 | 0.081864 |
| N | -2.90183 | -1.04019 | -0.01093 |
| C | -1.58618 | -1.25392 | -0.18388 |
| C | 1.559942 | 0.481392 | -1.36181 |
| C | 3.024785 | 0.324269 | -0.88665 |
| C | 2.969328 | -0.58409 | 0.347745 |
| H | 1.991829 | 0.568141 | 2.584914 |
| H | 0.285092 | 0.653709 | 2.093748 |
| H | 1.517678 | 1.593612 | 1.207519 |
| H | 0.853486 | -1.4862 | -0.85136 |
| H | -0.60736 | 1.972534 | -0.26469 |
| H | -3.04511 | 2.282493 | 0.066281 |
| H | -4.51301 | 0.237685 | 0.219833 |
| H | -1.27041 | -2.28589 | -0.2449 |
| H | 1.420427 | 0.204929 | -2.40577 |
| H | 1.227252 | 1.511703 | -1.24471 |
| H | 3.45306 | 1.294391 | -0.63761 |
| H | 3.654502 | -0.13228 | -1.6491 |
| H | 3.137304 | -1.62931 | 0.094763 |
| H | 3.633674 | -0.29824 | 1.158757 |
| H | -3.51699 | -1.84745 | 0.048989 |
| H | 1.299359 | -1.34426 | 1.345236 |
|  |  |  |  |
|  |  |  |  |
| Transition state |  |  |  |
| NIC-1 |  |  |  |
| C | -1.51402 | 0.453857 | 1.936869 |
| N | -1.36291 | -0.55873 | 0.884871 |
| C | -0.63824 | -0.01089 | -0.28473 |
| C | 0.849312 | 0.04796 | -0.05835 |
| C | 1.512453 | -0.80987 | 0.825526 |
| C | 2.899671 | -0.72788 | 0.948432 |
| C | 3.58765 | 0.212006 | 0.17821 |
| N | 2.977556 | 1.053956 | -0.66897 |
| C | 1.641663 | 0.965866 | -0.76554 |
| C | -1.03043 | -0.96163 | -1.45319 |
| C | -2.29401 | -1.70536 | -0.94992 |
| C | -2.67129 | -0.95365 | 0.330196 |
| H | -2.02538 | 1.349362 | 1.543598 |
| H | -0.52487 | 0.730677 | 2.322939 |
| H | -2.08694 | 0.021465 | 2.767849 |
| H | 0.917867 | -1.51259 | 1.401882 |
| H | 4.67114 | 0.300525 | 0.247703 |
| H | 1.159114 | 1.669897 | -1.44055 |
| H | -1.23954 | -0.36352 | -2.34416 |
| H | -0.22223 | -1.66318 | -1.69141 |
| H | -2.05573 | -2.75331 | -0.72849 |
| H | -3.10661 | -1.68815 | -1.68352 |
| H | -3.25962 | -0.05436 | 0.080297 |
| H | -3.20933 | -1.57967 | 1.055378 |
| H | 3.443554 | -1.37744 | 1.630055 |
| H | -1.04976 | 0.991794 | -0.52242 |
| O | -2.74357 | 2.009318 | -0.53236 |
| H | -3.07549 | 2.832717 | -0.9154 |
|  |  |  |  |
| TS-1 |  |  |  |
| C | -1.80644 | 0.378969 | 1.941152 |
| N | -1.63426 | -0.20858 | 0.649251 |
| C | -0.7178 | 0.315622 | -0.36227 |
| C | 0.71913 | 0.028209 | -0.15031 |
| C | 1.246908 | -0.5465 | 1.02416 |
| C | 2.624069 | -0.72034 | 1.162751 |
| C | 3.469064 | -0.32902 | 0.123928 |
| N | 3.004294 | 0.226974 | -1.01151 |
| C | 1.685139 | 0.401536 | -1.12185 |
| C | -1.30044 | -0.35192 | -1.61356 |
| C | -1.69371 | -1.77129 | -1.11591 |
| C | -2.01659 | -1.57872 | 0.390906 |
| H | -2.85045 | 0.262852 | 2.28156 |
| H | -1.58432 | 1.447949 | 1.864665 |
| H | -1.16503 | -0.05303 | 2.742143 |
| H | 0.572839 | -0.86251 | 1.812028 |
| H | 4.547931 | -0.4564 | 0.196431 |
| H | 1.346065 | 0.884837 | -2.03614 |
| H | -2.19332 | 0.197027 | -1.94084 |
| H | -0.60136 | -0.39511 | -2.45495 |
| H | -0.84034 | -2.44955 | -1.22961 |
| H | -2.53678 | -2.20492 | -1.66882 |
| H | -3.08758 | -1.72773 | 0.617229 |
| H | -1.46326 | -2.32293 | 1.004689 |
| H | 3.039773 | -1.16564 | 2.064479 |
| H | -0.9288 | 1.654949 | -0.39823 |
| O | -1.2284 | 2.890737 | -0.36501 |
| H | -2.16014 | 2.934653 | -0.62203 |
|  |  |  |  |
| NIC-2 |  |  |  |
| C | -1.78118 | 0.316915 | 1.993787 |
| N | -1.7346 | -0.14724 | 0.638959 |
| C | -0.71771 | 0.244197 | -0.33472 |
| C | 0.676733 | 0.008481 | -0.09785 |
| C | 1.253888 | -0.38947 | 1.147391 |
| C | 2.633755 | -0.50161 | 1.284138 |
| C | 3.471473 | -0.2444 | 0.195597 |
| N | 2.969866 | 0.124763 | -1.00503 |
| C | 1.653469 | 0.249434 | -1.12756 |
| C | -1.34539 | -0.27418 | -1.61705 |
| C | -1.98744 | -1.6348 | -1.18364 |
| C | -2.20911 | -1.49178 | 0.34663 |
| H | -2.82599 | 0.353792 | 2.349026 |
| H | -1.36686 | 1.328418 | 2.038699 |
| H | -1.22544 | -0.30812 | 2.724423 |
| H | 0.614405 | -0.62223 | 1.98935 |
| H | 4.553388 | -0.33092 | 0.271463 |
| H | 1.304293 | 0.584547 | -2.10322 |
| H | -2.13579 | 0.39793 | -1.98213 |
| H | -0.62674 | -0.41491 | -2.42967 |
| H | -1.29373 | -2.45799 | -1.38743 |
| H | -2.92326 | -1.84911 | -1.71695 |
| H | -3.26664 | -1.5984 | 0.638924 |
| H | -1.64355 | -2.27412 | 0.896105 |
| H | 3.063561 | -0.8082 | 2.237035 |
| H | -1.09757 | 2.072594 | -0.36728 |
| O | -1.59029 | 2.96032 | -0.33002 |
| H | -2.49233 | 2.6776 | -0.13448 |
|  |  |  |  |
| NIC-3 |  |  |  |
| C | -1.46276 | -1.82156 | 1.120405 |
| N | -1.70547 | -0.62058 | 0.318659 |
| C | -0.82612 | 0.237216 | -0.13208 |
| C | 0.618451 | 0.123156 | -0.01097 |
| C | 1.312145 | -1.09718 | -0.1413 |
| C | 2.695848 | -1.09191 | -0.04364 |
| C | 3.347138 | 0.125461 | 0.187147 |
| N | 2.705534 | 1.295129 | 0.312251 |
| C | 1.381606 | 1.292436 | 0.201251 |
| C | -1.51461 | 1.357633 | -0.8641 |
| C | -3.00294 | 1.188428 | -0.50179 |
| C | -3.10341 | -0.27905 | -0.06404 |
| H | -2.23532 | -1.87885 | 1.889627 |
| H | -0.4827 | -1.76529 | 1.591048 |
| H | -1.5258 | -2.71454 | 0.49165 |
| H | 0.785857 | -2.01859 | -0.36451 |
| H | 4.431106 | 0.164598 | 0.262277 |
| H | 0.892933 | 2.257 | 0.318274 |
| H | -1.10061 | 2.333729 | -0.60015 |
| H | -1.31713 | 1.220691 | -1.93608 |
| H | -3.66727 | 1.414553 | -1.33609 |
| H | -3.26601 | 1.846771 | 0.329711 |
| H | -3.75834 | -0.44655 | 0.793451 |
| H | -3.40714 | -0.95682 | -0.8698 |
| H | 3.266892 | -2.00656 | -0.158 |
|  |  |  |  |
| TS-2 |  |  |  |
| C | -1.69196 | -1.10295 | 1.369978 |
| N | -1.61654 | -1.13009 | -0.00804 |
| C | -0.80548 | 0.106806 | 0.300885 |
| C | 0.667422 | -0.01795 | 0.148087 |
| C | 1.258413 | -1.15732 | -0.41154 |
| C | 2.64005 | -1.16754 | -0.56939 |
| C | 3.371345 | -0.05099 | -0.14989 |
| N | 2.813708 | 1.038889 | 0.39739 |
| C | 1.491442 | 1.048675 | 0.541389 |
| C | -1.60271 | 1.287829 | -0.29454 |
| C | -3.04752 | 0.786988 | -0.40125 |
| C | -2.86712 | -0.70272 | -0.71639 |
| H | -2.62511 | -0.85486 | 1.884504 |
| H | -1.01652 | 0.251539 | 1.488374 |
| H | -0.91875 | -1.63082 | 1.928246 |
| H | 0.643059 | -1.99634 | -0.71886 |
| H | 4.453543 | -0.03174 | -0.25245 |
| H | 1.062692 | 1.946903 | 0.986485 |
| H | -1.48064 | 2.20915 | 0.276454 |
| H | -1.1623 | 1.441755 | -1.28534 |
| H | -3.60945 | 1.315576 | -1.17365 |
| H | -3.5832 | 0.935091 | 0.542895 |
| H | -3.69013 | -1.34273 | -0.39135 |
| H | -2.69489 | -0.8799 | -1.77953 |
| H | 3.145844 | -2.02221 | -1.00505 |
|  |  |  |  |
| NIC-4 |  |  |  |
| C | -1.32494 | -1.6069 | 1.377221 |
| N | -1.71079 | -0.64285 | 0.638095 |
| C | -0.85053 | 0.571945 | 0.289204 |
| C | 0.607571 | 0.233256 | 0.19344 |
| C | 1.136414 | -0.53418 | -0.85457 |
| C | 2.500375 | -0.80012 | -0.86439 |
| C | 3.28919 | -0.29414 | 0.176283 |
| N | 2.799095 | 0.434835 | 1.18465 |
| C | 1.488726 | 0.687495 | 1.182948 |
| C | -1.57267 | 1.118629 | -0.94767 |
| C | -3.0555 | 0.852809 | -0.64925 |
| C | -3.05107 | -0.54643 | -0.02396 |
| H | -1.99949 | -2.43152 | 1.591739 |
| H | -1.01273 | 1.257762 | 1.129715 |
| H | -0.31603 | -1.59369 | 1.781463 |
| H | 0.500718 | -0.91508 | -1.64943 |
| H | 4.359345 | -0.48594 | 0.198374 |
| H | 1.114879 | 1.293932 | 2.00883 |
| H | -1.34474 | 2.176381 | -1.08858 |
| H | -1.25008 | 0.583015 | -1.8461 |
| H | -3.68332 | 0.886145 | -1.54108 |
| H | -3.44284 | 1.586419 | 0.064122 |
| H | -3.83253 | -0.72536 | 0.716791 |
| H | -3.08673 | -1.33416 | -0.78241 |
| H | 2.950872 | -1.38414 | -1.65964 |
|  |  |  |  |
| NIC-5 |  |  |  |
| C | -1.01606 | -0.83954 | -1.89457 |
| N | -1.44481 | -0.07394 | -0.97148 |
| C | -0.58693 | 0.951151 | -0.24207 |
| C | 0.841732 | 0.515769 | -0.09515 |
| C | 1.207098 | -0.59665 | 0.678299 |
| C | 2.556749 | -0.91415 | 0.780824 |
| C | 3.489924 | -0.11572 | 0.108439 |
| N | 3.153605 | 0.940576 | -0.63984 |
| C | 1.855807 | 1.239831 | -0.73285 |
| C | -1.41036 | 1.199933 | 1.027616 |
| C | -2.86317 | 1.104193 | 0.539818 |
| C | -2.83622 | -0.08813 | -0.42186 |
| H | -1.69312 | -1.54117 | -2.3736 |
| H | 0.028412 | -0.78375 | -2.18863 |
| H | 0.449 | -1.19995 | 1.172047 |
| H | 4.552377 | -0.33939 | 0.172153 |
| H | 1.605958 | 2.109888 | -1.34102 |
| H | -1.19264 | 0.423925 | 1.765947 |
| H | -1.16131 | 2.169292 | 1.462197 |
| H | -3.15526 | 2.01847 | 0.01404 |
| H | -3.57544 | 0.941153 | 1.350378 |
| H | -2.9284 | -1.03593 | 0.115573 |
| H | -3.54977 | -0.03824 | -1.24609 |
| H | 2.889115 | -1.76179 | 1.371009 |
| H | -0.63673 | 1.841937 | -0.8805 |
| O | -1.61444 | -2.26728 | 1.476205 |
| H | -1.40907 | -3.19763 | 1.311106 |
| H | -1.91793 | -2.25183 | 2.394202 |
|  |  |  |  |
| TS-3 |  |  |  |
| C | -1.10015 | -1.34914 | -1.47653 |
| N | -1.51186 | -0.35771 | -0.6178 |
| C | -0.62753 | 0.723238 | -0.07746 |
| C | 0.82251 | 0.359814 | 0.046567 |
| C | 1.331427 | -0.43439 | 1.082861 |
| C | 2.696937 | -0.6933 | 1.110993 |
| C | 3.505397 | -0.1561 | 0.102909 |
| N | 3.034059 | 0.604004 | -0.89193 |
| C | 1.724318 | 0.852397 | -0.90651 |
| C | -1.41939 | 1.194393 | 1.143598 |
| C | -2.9116 | 1.014173 | 0.753309 |
| C | -2.91852 | 0.063041 | -0.46175 |
| H | -1.85896 | -1.67222 | -2.18812 |
| H | -0.07654 | -1.23177 | -1.82864 |
| H | 0.676979 | -0.85109 | 1.83841 |
| H | 4.576291 | -0.34501 | 0.09686 |
| H | 1.366321 | 1.487895 | -1.71819 |
| H | -1.15533 | 0.568419 | 1.999561 |
| H | -1.1679 | 2.226362 | 1.391573 |
| H | -3.37082 | 1.966639 | 0.48263 |
| H | -3.48959 | 0.594164 | 1.577929 |
| H | -3.53797 | -0.82793 | -0.36284 |
| H | -3.18401 | 0.56922 | -1.39951 |
| H | 3.133267 | -1.29962 | 1.897384 |
| H | -0.72183 | 1.485799 | -0.87168 |
| O | -1.4362 | -1.78918 | 0.765817 |
| H | -1.08032 | -2.20284 | -0.57449 |
| H | -1.65268 | -2.01942 | 1.695496 |
|  |  |  |  |
| NIC-6 |  |  |  |
| C | -1.14676 | -1.3091 | -1.65977 |
| N | -1.50237 | -0.65728 | -0.36872 |
| C | -0.64173 | 0.59882 | -0.02966 |
| C | 0.815694 | 0.298455 | 0.108292 |
| C | 1.383728 | -0.29319 | 1.245285 |
| C | 2.753062 | -0.53164 | 1.261668 |
| C | 3.510502 | -0.17165 | 0.141872 |
| N | 2.986461 | 0.401495 | -0.94818 |
| C | 1.673273 | 0.627594 | -0.95171 |
| C | -1.41805 | 1.18342 | 1.152129 |
| C | -2.91455 | 0.890402 | 0.853938 |
| C | -2.92421 | -0.14881 | -0.28872 |
| H | -1.26832 | -0.57532 | -2.45631 |
| H | -0.11053 | -1.63703 | -1.59068 |
| H | 0.779343 | -0.56384 | 2.104447 |
| H | 4.583189 | -0.34899 | 0.122624 |
| H | 1.273196 | 1.10818 | -1.84543 |
| H | -1.09525 | 0.727706 | 2.094059 |
| H | -1.20875 | 2.25024 | 1.243085 |
| H | -3.43775 | 1.791292 | 0.528979 |
| H | -3.438 | 0.521216 | 1.738315 |
| H | -3.57182 | -1.01307 | -0.13956 |
| H | -3.14682 | 0.301559 | -1.25778 |
| H | 3.230578 | -0.98578 | 2.123006 |
| H | -0.80155 | 1.226788 | -0.91326 |
| O | -1.30087 | -1.68148 | 0.598707 |
| H | -1.81284 | -2.15885 | -1.80275 |
| H | -1.59937 | -1.31752 | 1.45052 |
|  |  |  |  |
| NIC-7 |  |  |  |
| C | 1.599862 | 1.581613 | -0.32101 |
| N | 1.719751 | 0.193322 | -0.55339 |
| C | 0.669369 | -0.57901 | -1.24226 |
| C | -0.75492 | -0.17891 | -0.90216 |
| C | -1.27034 | -0.25884 | 0.400487 |
| C | -2.58157 | 0.146434 | 0.630761 |
| C | -3.34333 | 0.608389 | -0.44627 |
| N | -2.87523 | 0.689997 | -1.69576 |
| C | -1.60831 | 0.304512 | -1.89814 |
| C | 0.995572 | -2.0459 | -0.86025 |
| C | 2.448642 | -2.00791 | -0.36355 |
| C | 2.517532 | -0.64932 | 0.335067 |
| H | 1.164962 | 2.057612 | -1.2074 |
| H | -0.10087 | 1.860085 | 0.635896 |
| H | -0.65277 | -0.60076 | 1.226028 |
| H | -4.37219 | 0.929323 | -0.29602 |
| H | -1.24306 | 0.385175 | -2.92173 |
| H | 0.336447 | -2.36533 | -0.04744 |
| H | 0.834507 | -2.72704 | -1.69929 |
| H | 3.144552 | -2.02703 | -1.20878 |
| H | 2.689097 | -2.84275 | 0.299816 |
| H | 2.0988 | -0.70601 | 1.350007 |
| H | 3.538482 | -0.25444 | 0.406277 |
| H | -3.00629 | 0.106164 | 1.628427 |
| H | 0.789049 | -0.4421 | -2.32742 |
| O | 0.573871 | 0.191518 | 2.983541 |
| H | 1.301662 | 0.199584 | 3.616212 |
| H | 2.592035 | 2.005538 | -0.14165 |
| O | 0.834154 | 1.998421 | 0.84581 |
| H | 0.796743 | 0.892054 | 2.33779 |
|  |  |  |  |
| TS-4 |  |  |  |
| C | 1.561207 | 1.627591 | 0.080634 |
| N | 1.609562 | -0.04236 | 0.371048 |
| C | 0.572616 | -0.70434 | -0.53123 |
| C | -0.84973 | -0.33408 | -0.17642 |
| C | -1.66888 | -1.212 | 0.540662 |
| C | -2.98624 | -0.85299 | 0.809704 |
| C | -3.44018 | 0.379025 | 0.3426 |
| N | -2.67326 | 1.242832 | -0.3313 |
| C | -1.40274 | 0.900825 | -0.5871 |
| C | 0.998364 | -2.17093 | -0.48157 |
| C | 2.537038 | -2.10342 | -0.54454 |
| C | 2.897972 | -0.72617 | 0.061219 |
| H | 1.676609 | 1.641103 | -0.99206 |
| H | 0.585466 | 1.882978 | 0.45293 |
| H | -1.29317 | -2.17172 | 0.877778 |
| H | -4.46859 | 0.688597 | 0.520109 |
| H | -0.79638 | 1.683745 | -1.06672 |
| H | 0.685079 | -2.61933 | 0.463493 |
| H | 0.558929 | -2.74736 | -1.29805 |
| H | 2.88468 | -2.15817 | -1.57964 |
| H | 3.005001 | -2.92286 | 0.004103 |
| H | 3.468128 | -0.77092 | 0.987547 |
| H | 3.422448 | -0.08408 | -0.65001 |
| H | -3.64659 | -1.51474 | 1.360275 |
| H | 0.808107 | -0.29159 | -1.51853 |
| O | 1.308726 | -0.33319 | 1.732097 |
| H | 0.601749 | 0.305765 | 1.937627 |
| H | 2.399781 | 2.025068 | 0.644895 |
| O | 0.689545 | 3.352255 | -0.92041 |
| H | 0.459652 | 4.1337 | -0.39436 |
|  |  |  |  |
| NIC-8 |  |  |  |
| C | -0.04662 | 4.531048 | -0.29752 |
| N | 1.992139 | -1.23223 | 0.713837 |
| C | 1.03345 | -1.09398 | -0.41946 |
| C | -0.26726 | -0.44169 | -0.03273 |
| C | -1.25227 | -1.08802 | 0.723294 |
| C | -2.42539 | -0.40749 | 1.038984 |
| C | -2.58664 | 0.900406 | 0.585115 |
| N | -1.65862 | 1.532275 | -0.14187 |
| C | -0.53218 | 0.869702 | -0.43568 |
| C | 0.94567 | -2.49532 | -1.0746 |
| C | 2.315004 | -3.14776 | -0.75444 |
| C | 3.032351 | -2.10372 | 0.139217 |
| H | 0.732204 | 5.186295 | -0.70031 |
| H | 0.426836 | 3.852502 | 0.430049 |
| H | -1.0893 | -2.10203 | 1.067389 |
| H | -3.49036 | 1.461893 | 0.808882 |
| H | 0.188787 | 1.428492 | -1.02869 |
| H | 0.128347 | -3.07118 | -0.6355 |
| H | 0.748346 | -2.41343 | -2.14638 |
| H | 2.893931 | -3.36647 | -1.6556 |
| H | 2.177833 | -4.08701 | -0.21422 |
| H | 3.620186 | -2.53511 | 0.949427 |
| H | 3.685615 | -1.4573 | -0.45698 |
| H | -3.20576 | -0.88141 | 1.625153 |
| H | 1.550425 | -0.41901 | -1.11238 |
| O | 1.33048 | -2.0607 | 1.718653 |
| H | 1.276571 | -1.44973 | 2.46507 |
| H | -0.76325 | 5.162161 | 0.252517 |
| O | -0.63801 | 3.844928 | -1.38046 |
| H | -1.26672 | 3.2023 | -0.99588 |

Adsorption oriented structure

(Ⅰ)

| C | 1.242007 | 5.602266 | -0.00056 |
| --- | --- | --- | --- |
| C | 0.031942 | 4.915971 | -0.00091 |
| C | -1.16756 | 5.62923 | -0.00109 |
| C | 2.443501 | 4.901168 | -0.00016 |
| C | 0.024439 | 3.517321 | -0.00089 |
| C | 1.227023 | 2.812449 | -0.0004 |
| C | 2.436483 | 3.501371 | 0.000022 |
| C | 1.22962 | 1.416646 | -0.00025 |
| C | 0.052498 | 0.728933 | -0.00119 |
| C | -1.17427 | 1.421573 | -0.00158 |
| C | -1.18077 | 2.82161 | -0.0013 |
| C | -5.99543 | 5.635353 | -0.00033 |
| C | -7.20445 | 4.943274 | 0.000867 |
| C | -4.79339 | 4.933829 | -0.0008 |
| C | -7.21085 | 3.54902 | 0.000689 |
| C | -6.00809 | 2.845569 | -0.00055 |
| C | -4.79933 | 3.535965 | -0.00096 |
| C | -6.01405 | 1.450984 | -0.00124 |
| C | -7.24031 | 0.763663 | -0.00107 |
| C | -3.58757 | 5.631933 | -0.00115 |
| C | -2.38111 | 4.930513 | -0.00125 |
| C | -2.38671 | 3.525368 | -0.00137 |
| C | -3.59851 | 2.827849 | -0.00142 |
| C | -4.79425 | 0.747799 | -0.00203 |
| C | -3.6094 | 1.423657 | -0.00191 |
| C | -2.39207 | 0.719009 | -0.00218 |
| C | 6.066705 | 5.575067 | 0.0007 |
| C | 4.854415 | 4.889237 | 0.000501 |
| C | 3.651609 | 5.601032 | 0.000168 |
| C | 7.275739 | 4.860368 | 0.000716 |
| C | 4.848003 | 3.491373 | 0.000698 |
| C | 6.05426 | 2.794841 | 0.000922 |
| C | 7.269696 | 3.498964 | 0.00075 |
| C | 6.038741 | 1.393952 | 0.001349 |
| C | 4.85422 | 0.725554 | 0.001542 |
| C | 3.635449 | 1.428224 | 0.001033 |
| C | 3.6384 | 2.7904 | 0.000579 |
| H | 8.206266 | 5.388483 | 0.00067 |
| H | 8.195572 | 2.96273 | 0.000648 |
| C | 2.458898 | 0.735199 | 0.000843 |
| C | 0.021097 | -0.74401 | -7.9E-05 |
| C | 1.195331 | -1.43224 | 0.000103 |
| C | 1.18124 | -2.8232 | 0.000019 |
| C | -0.02882 | -3.5095 | -0.00033 |
| C | -1.22833 | -2.79624 | -0.00051 |
| C | 2.382733 | -3.5243 | 0.000418 |
| C | -0.03633 | -4.90815 | -0.00031 |
| C | 1.166256 | -5.61302 | 0.000178 |
| C | 2.375717 | -4.9241 | 0.0006 |
| C | -1.24154 | -5.60386 | -0.00072 |
| C | -7.26822 | -0.69638 | -0.00036 |
| C | -6.04872 | -1.3954 | -0.00049 |
| C | -6.0562 | -2.79012 | 0.00025 |
| C | -7.26521 | -3.48219 | 0.001445 |
| C | -4.85415 | -3.49164 | -0.00022 |
| C | -7.27161 | -4.87645 | 0.001267 |
| C | -6.06886 | -5.5799 | 0.000026 |
| C | -4.86009 | -4.8895 | -0.00038 |
| C | -4.82181 | -0.70441 | -0.00135 |
| C | -3.64834 | -2.79354 | -0.00057 |
| C | -2.44188 | -3.49496 | -0.00067 |
| C | -2.44748 | -4.9001 | -0.0008 |
| C | -3.65928 | -5.59762 | -0.00085 |
| C | -3.6451 | -1.39403 | -0.00086 |
| C | -1.20938 | -1.43392 | -0.00045 |
| C | -2.41785 | -0.71165 | -0.00062 |
| C | 4.824627 | -0.77027 | 0.001102 |
| C | 6.003031 | -1.44938 | 0.00139 |
| C | 6.005939 | -2.8504 | 0.001278 |
| C | 4.793648 | -3.53623 | 0.001079 |
| C | 3.590841 | -2.82444 | 0.000746 |
| C | 3.59981 | -1.46205 | 0.000743 |
| C | 7.214972 | -3.5651 | 0.001294 |
| C | 4.787236 | -4.9341 | 0.001276 |
| C | 5.993493 | -5.63063 | 0.0015 |
| C | 7.208929 | -4.9265 | 0.001328 |
| C | 3.577634 | -5.63507 | 0.001157 |
| H | 8.1455 | -3.03699 | 0.001248 |
| H | 8.134806 | -5.46274 | 0.001226 |
| C | 2.428822 | -0.75917 | 0.000472 |
| C | 7.179882 | -0.76759 | 0.001729 |
| C | 7.209341 | 0.701483 | 0.001416 |
| H | 8.102225 | -1.30997 | 0.002158 |
| H | 8.143401 | 1.223432 | 0.001315 |
| H | 5.994249 | -6.70063 | 0.001802 |
| H | 3.572843 | -6.70506 | 0.001489 |
| H | 1.15963 | -6.683 | 0.000231 |
| H | -1.24407 | -6.67386 | -0.00097 |
| H | -3.66491 | -6.66761 | -0.00124 |
| H | -6.07435 | -6.64989 | -0.00061 |
| H | -8.20098 | -5.40671 | 0.002084 |
| H | -8.18964 | -2.94336 | 0.0025 |
| H | -8.19912 | -1.22395 | 0.000244 |
| H | -8.15832 | 1.31335 | -0.00147 |
| H | -8.14021 | 3.018756 | 0.001506 |
| H | -8.12887 | 5.482109 | 0.001922 |
| H | -5.99095 | 6.705344 | -0.00089 |
| H | -1.16004 | 6.699204 | -0.00111 |
| H | -3.5866 | 6.701932 | -0.00135 |
| H | 1.248586 | 6.672246 | -0.0006 |
| H | 3.656577 | 6.671021 | 0.000164 |
| H | 6.076888 | 6.645019 | 0.00084 |
| C | 1.091472 | -1.42058 | 4.345046 |
| N | 1.309951 | -0.81545 | 3.028481 |
| C | 0.562939 | 0.447682 | 2.827387 |
| C | -0.9184 | 0.327366 | 3.092092 |
| C | -1.64463 | 1.260675 | 3.832244 |
| C | -3.01883 | 1.082731 | 3.995304 |
| C | -3.62082 | -0.0291 | 3.411689 |
| N | -2.94697 | -0.94003 | 2.695112 |
| C | -1.63353 | -0.75034 | 2.545735 |
| C | 1.349718 | 1.528536 | 3.611972 |
| C | 2.815577 | 1.031999 | 3.529914 |
| C | 2.700722 | -0.37157 | 2.861105 |
| H | 1.707331 | -2.32202 | 4.417781 |
| H | 0.045383 | -1.71802 | 4.446089 |
| H | 1.342333 | -0.76684 | 5.199012 |
| H | 0.682761 | 0.679237 | 1.757493 |
| H | -1.14975 | 2.120068 | 4.273804 |
| H | -3.6104 | 1.792865 | 4.565303 |
| H | -4.69044 | -0.20009 | 3.519436 |
| H | -1.09738 | -1.50051 | 1.967609 |
| H | 1.211597 | 2.526577 | 3.185313 |
| H | 1.012312 | 1.569662 | 4.652884 |
| H | 3.262546 | 0.963956 | 4.526168 |
| H | 3.447582 | 1.702038 | 2.93931 |
| H | 2.906934 | -0.29022 | 1.786769 |
| H | 3.390391 | -1.11093 | 3.276608 |

(Ⅱ)

| C | -1.40124 | -5.62693 | -0.48139 |
| --- | --- | --- | --- |
| C | -0.16518 | -4.98869 | -0.47352 |
| C | 1.005086 | -5.7483 | -0.44142 |
| C | -2.57398 | -4.87939 | -0.5131 |
| C | -0.10247 | -3.59164 | -0.49761 |
| C | -1.27615 | -2.84029 | -0.52931 |
| C | -2.51172 | -3.48117 | -0.53705 |
| C | -1.22365 | -1.44568 | -0.55338 |
| C | -0.02044 | -0.80475 | -0.54667 |
| C | 1.177882 | -1.54482 | -0.51468 |
| C | 1.129112 | -2.94381 | -0.49004 |
| C | 5.828329 | -5.94359 | -0.36032 |
| C | 7.063545 | -5.29951 | -0.35103 |
| C | 4.65507 | -5.19562 | -0.39292 |
| C | 7.124979 | -3.9068 | -0.37525 |
| C | 5.951098 | -3.1569 | -0.40866 |
| C | 4.716185 | -3.79929 | -0.4172 |
| C | 6.012108 | -1.76385 | -0.4334 |
| C | 7.264387 | -1.12522 | -0.42476 |
| C | 3.422798 | -5.84583 | -0.40122 |
| C | 2.245117 | -5.09779 | -0.43351 |
| C | 2.30618 | -3.69417 | -0.45788 |
| C | 3.544417 | -3.04479 | -0.44988 |
| C | 4.821191 | -1.01354 | -0.46664 |
| C | 3.61073 | -1.64233 | -0.47451 |
| C | 2.422326 | -0.89064 | -0.5072 |
| C | -6.22052 | -5.41067 | -0.56078 |
| C | -4.98225 | -4.77298 | -0.55271 |
| C | -3.80863 | -5.53126 | -0.52073 |
| C | -7.40024 | -4.64926 | -0.59323 |
| C | -4.92067 | -3.37667 | -0.57662 |
| C | -6.09834 | -2.63351 | -0.6085 |
| C | -7.34047 | -3.28935 | -0.61668 |
| C | -6.02755 | -1.23452 | -0.63208 |
| C | -4.81772 | -0.61316 | -0.62379 |
| C | -3.62778 | -1.36295 | -0.59187 |
| C | -3.68449 | -2.72375 | -0.56878 |
| H | -8.35077 | -5.14042 | -0.59959 |
| H | -8.24434 | -2.71734 | -0.64145 |
| C | -2.42494 | -0.71667 | -0.58451 |
| C | 0.069061 | 0.665618 | -0.57055 |
| C | -1.07695 | 1.399219 | -0.6018 |
| C | -1.00796 | 2.78834 | -0.62574 |
| C | 0.228104 | 3.426574 | -0.61787 |
| C | 1.398369 | 2.666969 | -0.58576 |
| C | -2.1807 | 3.535876 | -0.65745 |
| C | 0.29081 | 4.823629 | -0.64196 |
| C | -0.88287 | 5.57498 | -0.67366 |
| C | -2.11843 | 4.9341 | -0.6814 |
| C | 1.522395 | 5.471459 | -0.63438 |
| C | 7.3499 | 0.332388 | -0.44888 |
| C | 6.1591 | 1.078543 | -0.48137 |
| C | 6.221612 | 2.47168 | -0.50467 |
| C | 7.456829 | 3.115761 | -0.49538 |
| C | 5.048353 | 3.219648 | -0.53727 |
| C | 7.518262 | 4.508466 | -0.5196 |
| C | 6.344381 | 5.258372 | -0.55301 |
| C | 5.109468 | 4.615976 | -0.56155 |
| C | 4.906031 | 0.436257 | -0.49066 |
| C | 3.816081 | 2.569438 | -0.54557 |
| C | 2.6384 | 3.31748 | -0.57786 |
| C | 2.699465 | 4.721097 | -0.60223 |
| C | 3.937702 | 5.370481 | -0.59423 |
| C | 3.757604 | 1.171353 | -0.52167 |
| C | 1.325664 | 1.306663 | -0.56242 |
| C | 2.504533 | 0.537706 | -0.53 |
| C | -4.7291 | 0.880102 | -0.64964 |
| C | -5.87963 | 1.604763 | -0.6807 |
| C | -5.82723 | 3.004597 | -0.70513 |
| C | -4.58897 | 3.642281 | -0.69706 |
| C | -3.41534 | 2.884009 | -0.66508 |
| C | -3.47808 | 1.523244 | -0.64163 |
| C | -7.00696 | 3.76601 | -0.73758 |
| C | -4.52738 | 5.038599 | -0.72097 |
| C | -5.70506 | 5.781756 | -0.75285 |
| C | -6.94718 | 5.125912 | -0.76103 |
| C | -3.29121 | 5.691518 | -0.71313 |
| H | -7.95749 | 3.27485 | -0.74394 |
| H | -7.85105 | 5.697927 | -0.7858 |
| C | -2.33589 | 0.775118 | -0.61027 |
| C | -7.08234 | 0.969728 | -0.68811 |
| C | -7.16975 | -0.49683 | -0.66346 |
| H | -7.98244 | 1.547761 | -0.7124 |
| H | -8.12357 | -0.98169 | -0.67004 |
| H | -5.66358 | 6.850797 | -0.7711 |
| H | -3.24419 | 6.760331 | -0.73125 |
| H | -0.83401 | 6.643706 | -0.69203 |
| H | 1.567163 | 6.540358 | -0.65313 |
| H | 3.985567 | 6.439245 | -0.61306 |
| H | 6.392118 | 6.327137 | -0.57209 |
| H | 8.467704 | 5.001832 | -0.51252 |
| H | 8.359127 | 2.541221 | -0.46963 |
| H | 8.300769 | 0.822998 | -0.44194 |
| H | 8.159875 | -1.71038 | -0.40038 |
| H | 8.07442 | -3.41343 | -0.36817 |
| H | 7.965843 | -5.87405 | -0.32528 |
| H | 5.781624 | -7.01242 | -0.34242 |
| H | 0.955339 | -6.81698 | -0.42302 |
| H | 3.37959 | -6.9148 | -0.3829 |
| H | -1.45005 | -6.69565 | -0.46301 |
| H | -3.85582 | -6.60006 | -0.50228 |
| H | -6.27293 | -6.47922 | -0.54227 |
| C | -1.25977 | -1.1447 | 2.051461 |
| N | -1.47327 | -1.11546 | 3.498893 |
| C | -0.89922 | 0.079354 | 4.154653 |
| C | 0.576589 | 0.278552 | 3.930676 |
| C | 1.148032 | 1.529848 | 3.699357 |
| C | 2.535664 | 1.63964 | 3.607893 |
| C | 3.306428 | 0.487945 | 3.745701 |
| N | 2.782097 | -0.72755 | 3.961398 |
| C | 1.452144 | -0.81261 | 4.052334 |
| C | -1.8574 | 1.243671 | 3.818217 |
| C | -3.23959 | 0.546023 | 3.803963 |
| C | -2.89799 | -0.97881 | 3.842789 |
| H | -1.72023 | -2.05211 | 1.648291 |
| H | -0.18803 | -1.18711 | 1.840996 |
| H | -1.67864 | -0.28001 | 1.506583 |
| H | -1.02267 | -0.1196 | 5.22266 |
| H | 0.522758 | 2.413346 | 3.61938 |
| H | 3.010512 | 2.603343 | 3.451805 |
| H | 4.392225 | 0.539453 | 3.695997 |
| H | 1.0392 | -1.80142 | 4.239969 |
| H | -1.79924 | 2.053324 | 4.549485 |
| H | -1.61788 | 1.66958 | 2.837597 |
| H | -3.81027 | 0.811125 | 2.909027 |
| H | -3.8442 | 0.831151 | 4.669958 |
| H | -3.04348 | -1.3659 | 4.858049 |
| H | -3.51167 | -1.5795 | 3.166138 |

(Ⅲ)

| C | -1.40124 | -5.62693 | -0.48139 |
| --- | --- | --- | --- |
| C | -0.16518 | -4.98869 | -0.47352 |
| C | 1.005086 | -5.7483 | -0.44142 |
| C | -2.57398 | -4.87939 | -0.5131 |
| C | -0.10247 | -3.59164 | -0.49761 |
| C | -1.27615 | -2.84029 | -0.52931 |
| C | -2.51172 | -3.48117 | -0.53705 |
| C | -1.22365 | -1.44568 | -0.55338 |
| C | -0.02044 | -0.80475 | -0.54667 |
| C | 1.177882 | -1.54482 | -0.51468 |
| C | 1.129112 | -2.94381 | -0.49004 |
| C | 5.828329 | -5.94359 | -0.36032 |
| C | 7.063545 | -5.29951 | -0.35103 |
| C | 4.65507 | -5.19562 | -0.39292 |
| C | 7.124979 | -3.9068 | -0.37525 |
| C | 5.951098 | -3.1569 | -0.40866 |
| C | 4.716185 | -3.79929 | -0.4172 |
| C | 6.012108 | -1.76385 | -0.4334 |
| C | 7.264387 | -1.12522 | -0.42476 |
| C | 3.422798 | -5.84583 | -0.40122 |
| C | 2.245117 | -5.09779 | -0.43351 |
| C | 2.30618 | -3.69417 | -0.45788 |
| C | 3.544417 | -3.04479 | -0.44988 |
| C | 4.821191 | -1.01354 | -0.46664 |
| C | 3.61073 | -1.64233 | -0.47451 |
| C | 2.422326 | -0.89064 | -0.5072 |
| C | -6.22052 | -5.41067 | -0.56078 |
| C | -4.98225 | -4.77298 | -0.55271 |
| C | -3.80863 | -5.53126 | -0.52073 |
| C | -7.40024 | -4.64926 | -0.59323 |
| C | -4.92067 | -3.37667 | -0.57662 |
| C | -6.09834 | -2.63351 | -0.6085 |
| C | -7.34047 | -3.28935 | -0.61668 |
| C | -6.02755 | -1.23452 | -0.63208 |
| C | -4.81772 | -0.61316 | -0.62379 |
| C | -3.62778 | -1.36295 | -0.59187 |
| C | -3.68449 | -2.72375 | -0.56878 |
| H | -8.35077 | -5.14042 | -0.59959 |
| H | -8.24434 | -2.71734 | -0.64145 |
| C | -2.42494 | -0.71667 | -0.58451 |
| C | 0.069061 | 0.665618 | -0.57055 |
| C | -1.07695 | 1.399219 | -0.6018 |
| C | -1.00796 | 2.78834 | -0.62574 |
| C | 0.228104 | 3.426574 | -0.61787 |
| C | 1.398369 | 2.666969 | -0.58576 |
| C | -2.1807 | 3.535876 | -0.65745 |
| C | 0.29081 | 4.823629 | -0.64196 |
| C | -0.88287 | 5.57498 | -0.67366 |
| C | -2.11843 | 4.9341 | -0.6814 |
| C | 1.522395 | 5.471459 | -0.63438 |
| C | 7.3499 | 0.332388 | -0.44888 |
| C | 6.1591 | 1.078543 | -0.48137 |
| C | 6.221612 | 2.47168 | -0.50467 |
| C | 7.456829 | 3.115761 | -0.49538 |
| C | 5.048353 | 3.219648 | -0.53727 |
| C | 7.518262 | 4.508466 | -0.5196 |
| C | 6.344381 | 5.258372 | -0.55301 |
| C | 5.109468 | 4.615976 | -0.56155 |
| C | 4.906031 | 0.436257 | -0.49066 |
| C | 3.816081 | 2.569438 | -0.54557 |
| C | 2.6384 | 3.31748 | -0.57786 |
| C | 2.699465 | 4.721097 | -0.60223 |
| C | 3.937702 | 5.370481 | -0.59423 |
| C | 3.757604 | 1.171353 | -0.52167 |
| C | 1.325664 | 1.306663 | -0.56242 |
| C | 2.504533 | 0.537706 | -0.53 |
| C | -4.7291 | 0.880102 | -0.64964 |
| C | -5.87963 | 1.604763 | -0.6807 |
| C | -5.82723 | 3.004597 | -0.70513 |
| C | -4.58897 | 3.642281 | -0.69706 |
| C | -3.41534 | 2.884009 | -0.66508 |
| C | -3.47808 | 1.523244 | -0.64163 |
| C | -7.00696 | 3.76601 | -0.73758 |
| C | -4.52738 | 5.038599 | -0.72097 |
| C | -5.70506 | 5.781756 | -0.75285 |
| C | -6.94718 | 5.125912 | -0.76103 |
| C | -3.29121 | 5.691518 | -0.71313 |
| H | -7.95749 | 3.27485 | -0.74394 |
| H | -7.85105 | 5.697927 | -0.7858 |
| C | -2.33589 | 0.775118 | -0.61027 |
| C | -7.08234 | 0.969728 | -0.68811 |
| C | -7.16975 | -0.49683 | -0.66346 |
| H | -7.98244 | 1.547761 | -0.7124 |
| H | -8.12357 | -0.98169 | -0.67004 |
| H | -5.66358 | 6.850797 | -0.7711 |
| H | -3.24419 | 6.760331 | -0.73125 |
| H | -0.83401 | 6.643706 | -0.69203 |
| H | 1.567163 | 6.540358 | -0.65313 |
| H | 3.985567 | 6.439245 | -0.61306 |
| H | 6.392118 | 6.327137 | -0.57209 |
| H | 8.467704 | 5.001832 | -0.51252 |
| H | 8.359127 | 2.541221 | -0.46963 |
| H | 8.300769 | 0.822998 | -0.44194 |
| H | 8.159875 | -1.71038 | -0.40038 |
| H | 8.07442 | -3.41343 | -0.36817 |
| H | 7.965843 | -5.87405 | -0.32528 |
| H | 5.781624 | -7.01242 | -0.34242 |
| H | 0.955339 | -6.81698 | -0.42302 |
| H | 3.37959 | -6.9148 | -0.3829 |
| H | -1.45005 | -6.69565 | -0.46301 |
| H | -3.85582 | -6.60006 | -0.50228 |
| H | -6.27293 | -6.47922 | -0.54227 |
| C | -1.34195 | -1.41134 | 4.723511 |
| N | -1.55299 | 0.036691 | 4.73666 |
| C | -0.9021 | 0.736775 | 3.608229 |
| C | 0.583552 | 0.516849 | 3.497201 |
| C | 1.235473 | 0.33272 | 2.277819 |
| C | 2.627307 | 0.241972 | 2.255334 |
| C | 3.321227 | 0.333029 | 3.45935 |
| N | 2.718654 | 0.502812 | 4.645497 |
| C | 1.385991 | 0.593842 | 4.647064 |
| C | -1.78223 | 0.448217 | 2.371681 |
| C | -3.20703 | 0.410527 | 2.976573 |
| C | -2.96572 | 0.38933 | 4.520804 |
| H | -1.86071 | -1.84818 | 5.582602 |
| H | -0.27528 | -1.62608 | 4.827556 |
| H | -1.7035 | -1.9212 | 3.812823 |
| H | -1.03821 | 1.796586 | 3.840041 |
| H | 0.669222 | 0.288677 | 1.352966 |
| H | 3.164063 | 0.122121 | 1.319334 |
| H | 4.408066 | 0.282534 | 3.476908 |
| H | 0.909369 | 0.744082 | 5.613369 |
| H | -1.67126 | 1.210165 | 1.596571 |
| H | -1.51546 | -0.51578 | 1.924536 |
| H | -3.75923 | -0.47197 | 2.640233 |
| H | -3.79166 | 1.288494 | 2.686461 |
| H | -3.13611 | 1.389197 | 4.936726 |
| H | -3.61737 | -0.3085 | 5.053424 |

(Ⅳ)

| C | -1.40124 | -5.62693 | -0.48139 |
| --- | --- | --- | --- |
| C | -0.16518 | -4.98869 | -0.47352 |
| C | 1.005086 | -5.7483 | -0.44142 |
| C | -2.57398 | -4.87939 | -0.5131 |
| C | -0.10247 | -3.59164 | -0.49761 |
| C | -1.27615 | -2.84029 | -0.52931 |
| C | -2.51172 | -3.48117 | -0.53705 |
| C | -1.22365 | -1.44568 | -0.55338 |
| C | -0.02044 | -0.80475 | -0.54667 |
| C | 1.177882 | -1.54482 | -0.51468 |
| C | 1.129112 | -2.94381 | -0.49004 |
| C | 5.828329 | -5.94359 | -0.36032 |
| C | 7.063545 | -5.29951 | -0.35103 |
| C | 4.65507 | -5.19562 | -0.39292 |
| C | 7.124979 | -3.9068 | -0.37525 |
| C | 5.951098 | -3.1569 | -0.40866 |
| C | 4.716185 | -3.79929 | -0.4172 |
| C | 6.012108 | -1.76385 | -0.4334 |
| C | 7.264387 | -1.12522 | -0.42476 |
| C | 3.422798 | -5.84583 | -0.40122 |
| C | 2.245117 | -5.09779 | -0.43351 |
| C | 2.30618 | -3.69417 | -0.45788 |
| C | 3.544417 | -3.04479 | -0.44988 |
| C | 4.821191 | -1.01354 | -0.46664 |
| C | 3.61073 | -1.64233 | -0.47451 |
| C | 2.422326 | -0.89064 | -0.5072 |
| C | -6.22052 | -5.41067 | -0.56078 |
| C | -4.98225 | -4.77298 | -0.55271 |
| C | -3.80863 | -5.53126 | -0.52073 |
| C | -7.40024 | -4.64926 | -0.59323 |
| C | -4.92067 | -3.37667 | -0.57662 |
| C | -6.09834 | -2.63351 | -0.6085 |
| C | -7.34047 | -3.28935 | -0.61668 |
| C | -6.02755 | -1.23452 | -0.63208 |
| C | -4.81772 | -0.61316 | -0.62379 |
| C | -3.62778 | -1.36295 | -0.59187 |
| C | -3.68449 | -2.72375 | -0.56878 |
| H | -8.35077 | -5.14042 | -0.59959 |
| H | -8.24434 | -2.71734 | -0.64145 |
| C | -2.42494 | -0.71667 | -0.58451 |
| C | 0.069061 | 0.665618 | -0.57055 |
| C | -1.07695 | 1.399219 | -0.6018 |
| C | -1.00796 | 2.78834 | -0.62574 |
| C | 0.228104 | 3.426574 | -0.61787 |
| C | 1.398369 | 2.666969 | -0.58576 |
| C | -2.1807 | 3.535876 | -0.65745 |
| C | 0.29081 | 4.823629 | -0.64196 |
| C | -0.88287 | 5.57498 | -0.67366 |
| C | -2.11843 | 4.9341 | -0.6814 |
| C | 1.522395 | 5.471459 | -0.63438 |
| C | 7.3499 | 0.332388 | -0.44888 |
| C | 6.1591 | 1.078543 | -0.48137 |
| C | 6.221612 | 2.47168 | -0.50467 |
| C | 7.456829 | 3.115761 | -0.49538 |
| C | 5.048353 | 3.219648 | -0.53727 |
| C | 7.518262 | 4.508466 | -0.5196 |
| C | 6.344381 | 5.258372 | -0.55301 |
| C | 5.109468 | 4.615976 | -0.56155 |
| C | 4.906031 | 0.436257 | -0.49066 |
| C | 3.816081 | 2.569438 | -0.54557 |
| C | 2.6384 | 3.31748 | -0.57786 |
| C | 2.699465 | 4.721097 | -0.60223 |
| C | 3.937702 | 5.370481 | -0.59423 |
| C | 3.757604 | 1.171353 | -0.52167 |
| C | 1.325664 | 1.306663 | -0.56242 |
| C | 2.504533 | 0.537706 | -0.53 |
| C | -4.7291 | 0.880102 | -0.64964 |
| C | -5.87963 | 1.604763 | -0.6807 |
| C | -5.82723 | 3.004597 | -0.70513 |
| C | -4.58897 | 3.642281 | -0.69706 |
| C | -3.41534 | 2.884009 | -0.66508 |
| C | -3.47808 | 1.523244 | -0.64163 |
| C | -7.00696 | 3.76601 | -0.73758 |
| C | -4.52738 | 5.038599 | -0.72097 |
| C | -5.70506 | 5.781756 | -0.75285 |
| C | -6.94718 | 5.125912 | -0.76103 |
| C | -3.29121 | 5.691518 | -0.71313 |
| H | -7.95749 | 3.27485 | -0.74394 |
| H | -7.85105 | 5.697927 | -0.7858 |
| C | -2.33589 | 0.775118 | -0.61027 |
| C | -7.08234 | 0.969728 | -0.68811 |
| C | -7.16975 | -0.49683 | -0.66346 |
| H | -7.98244 | 1.547761 | -0.7124 |
| H | -8.12357 | -0.98169 | -0.67004 |
| H | -5.66358 | 6.850797 | -0.7711 |
| H | -3.24419 | 6.760331 | -0.73125 |
| H | -0.83401 | 6.643706 | -0.69203 |
| H | 1.567163 | 6.540358 | -0.65313 |
| H | 3.985567 | 6.439245 | -0.61306 |
| H | 6.392118 | 6.327137 | -0.57209 |
| H | 8.467704 | 5.001832 | -0.51252 |
| H | 8.359127 | 2.541221 | -0.46963 |
| H | 8.300769 | 0.822998 | -0.44194 |
| H | 8.159875 | -1.71038 | -0.40038 |
| H | 8.07442 | -3.41343 | -0.36817 |
| H | 7.965843 | -5.87405 | -0.32528 |
| H | 5.781624 | -7.01242 | -0.34242 |
| H | 0.955339 | -6.81698 | -0.42302 |
| H | 3.37959 | -6.9148 | -0.3829 |
| H | -1.45005 | -6.69565 | -0.46301 |
| H | -3.85582 | -6.60006 | -0.50228 |
| H | -6.27293 | -6.47922 | -0.54227 |
| C | -1.0682 | 1.740471 | 2.296336 |
| N | -1.48874 | 0.339177 | 2.328273 |
| C | -0.98377 | -0.40036 | 3.505235 |
| C | 0.513769 | -0.39411 | 3.663894 |
| C | 1.146488 | -0.25594 | 4.899358 |
| C | 2.535158 | -0.36746 | 4.970799 |
| C | 3.246202 | -0.60847 | 3.797852 |
| N | 2.663318 | -0.73824 | 2.596907 |
| C | 1.332442 | -0.63472 | 2.548589 |
| C | -1.851 | 0.064284 | 4.696442 |
| C | -3.23489 | 0.283267 | 4.037457 |
| C | -2.94405 | 0.204783 | 2.503645 |
| H | -1.48969 | 2.211823 | 1.402943 |
| H | 0.021187 | 1.793475 | 2.224662 |
| H | -1.37989 | 2.334963 | 3.173522 |
| H | -1.26635 | -1.43789 | 3.307417 |
| H | 0.563662 | -0.0916 | 5.800045 |
| H | 3.053614 | -0.28792 | 5.921295 |
| H | 4.328702 | -0.71714 | 3.819315 |
| H | 0.869948 | -0.7543 | 1.571201 |
| H | -1.87768 | -0.67271 | 5.502543 |
| H | -1.46016 | 0.997105 | 5.117715 |
| H | -3.66216 | 1.249862 | 4.320281 |
| H | -3.95087 | -0.4876 | 4.337332 |
| H | -3.24587 | -0.7762 | 2.118488 |
| H | -3.46905 | 0.967038 | 1.921728 |
| (Ⅴ) |  |  |  |
| C | -1.40124 | -5.62693 | -0.48139 |
| C | -0.16518 | -4.98869 | -0.47352 |
| C | 1.005086 | -5.7483 | -0.44142 |
| C | -2.57398 | -4.87939 | -0.5131 |
| C | -0.10247 | -3.59164 | -0.49761 |
| C | -1.27615 | -2.84029 | -0.52931 |
| C | -2.51172 | -3.48117 | -0.53705 |
| C | -1.22365 | -1.44568 | -0.55338 |
| C | -0.02044 | -0.80475 | -0.54667 |
| C | 1.177882 | -1.54482 | -0.51468 |
| C | 1.129112 | -2.94381 | -0.49004 |
| C | 5.828329 | -5.94359 | -0.36032 |
| C | 7.063545 | -5.29951 | -0.35103 |
| C | 4.65507 | -5.19562 | -0.39292 |
| C | 7.124979 | -3.9068 | -0.37525 |
| C | 5.951098 | -3.1569 | -0.40866 |
| C | 4.716185 | -3.79929 | -0.4172 |
| C | 6.012108 | -1.76385 | -0.4334 |
| C | 7.264387 | -1.12522 | -0.42476 |
| C | 3.422798 | -5.84583 | -0.40122 |
| C | 2.245117 | -5.09779 | -0.43351 |
| C | 2.30618 | -3.69417 | -0.45788 |
| C | 3.544417 | -3.04479 | -0.44988 |
| C | 4.821191 | -1.01354 | -0.46664 |
| C | 3.61073 | -1.64233 | -0.47451 |
| C | 2.422326 | -0.89064 | -0.5072 |
| C | -6.22052 | -5.41067 | -0.56078 |
| C | -4.98225 | -4.77298 | -0.55271 |
| C | -3.80863 | -5.53126 | -0.52073 |
| C | -7.40024 | -4.64926 | -0.59323 |
| C | -4.92067 | -3.37667 | -0.57662 |
| C | -6.09834 | -2.63351 | -0.6085 |
| C | -7.34047 | -3.28935 | -0.61668 |
| C | -6.02755 | -1.23452 | -0.63208 |
| C | -4.81772 | -0.61316 | -0.62379 |
| C | -3.62778 | -1.36295 | -0.59187 |
| C | -3.68449 | -2.72375 | -0.56878 |
| H | -8.35077 | -5.14042 | -0.59959 |
| H | -8.24434 | -2.71734 | -0.64145 |
| C | -2.42494 | -0.71667 | -0.58451 |
| C | 0.069061 | 0.665618 | -0.57055 |
| C | -1.07695 | 1.399219 | -0.6018 |
| C | -1.00796 | 2.78834 | -0.62574 |
| C | 0.228104 | 3.426574 | -0.61787 |
| C | 1.398369 | 2.666969 | -0.58576 |
| C | -2.1807 | 3.535876 | -0.65745 |
| C | 0.29081 | 4.823629 | -0.64196 |
| C | -0.88287 | 5.57498 | -0.67366 |
| C | -2.11843 | 4.9341 | -0.6814 |
| C | 1.522395 | 5.471459 | -0.63438 |
| C | 7.3499 | 0.332388 | -0.44888 |
| C | 6.1591 | 1.078543 | -0.48137 |
| C | 6.221612 | 2.47168 | -0.50467 |
| C | 7.456829 | 3.115761 | -0.49538 |
| C | 5.048353 | 3.219648 | -0.53727 |
| C | 7.518262 | 4.508466 | -0.5196 |
| C | 6.344381 | 5.258372 | -0.55301 |
| C | 5.109468 | 4.615976 | -0.56155 |
| C | 4.906031 | 0.436257 | -0.49066 |
| C | 3.816081 | 2.569438 | -0.54557 |
| C | 2.6384 | 3.31748 | -0.57786 |
| C | 2.699465 | 4.721097 | -0.60223 |
| C | 3.937702 | 5.370481 | -0.59423 |
| C | 3.757604 | 1.171353 | -0.52167 |
| C | 1.325664 | 1.306663 | -0.56242 |
| C | 2.504533 | 0.537706 | -0.53 |
| C | -4.7291 | 0.880102 | -0.64964 |
| C | -5.87963 | 1.604763 | -0.6807 |
| C | -5.82723 | 3.004597 | -0.70513 |
| C | -4.58897 | 3.642281 | -0.69706 |
| C | -3.41534 | 2.884009 | -0.66508 |
| C | -3.47808 | 1.523244 | -0.64163 |
| C | -7.00696 | 3.76601 | -0.73758 |
| C | -4.52738 | 5.038599 | -0.72097 |
| C | -5.70506 | 5.781756 | -0.75285 |
| C | -6.94718 | 5.125912 | -0.76103 |
| C | -3.29121 | 5.691518 | -0.71313 |
| H | -7.95749 | 3.27485 | -0.74394 |
| H | -7.85105 | 5.697927 | -0.7858 |
| C | -2.33589 | 0.775118 | -0.61027 |
| C | -7.08234 | 0.969728 | -0.68811 |
| C | -7.16975 | -0.49683 | -0.66346 |
| H | -7.98244 | 1.547761 | -0.7124 |
| H | -8.12357 | -0.98169 | -0.67004 |
| H | -5.66358 | 6.850797 | -0.7711 |
| H | -3.24419 | 6.760331 | -0.73125 |
| H | -0.83401 | 6.643706 | -0.69203 |
| H | 1.567163 | 6.540358 | -0.65313 |
| H | 3.985567 | 6.439245 | -0.61306 |
| H | 6.392118 | 6.327137 | -0.57209 |
| H | 8.467704 | 5.001832 | -0.51252 |
| H | 8.359127 | 2.541221 | -0.46963 |
| H | 8.300769 | 0.822998 | -0.44194 |
| H | 8.159875 | -1.71038 | -0.40038 |
| H | 8.07442 | -3.41343 | -0.36817 |
| H | 7.965843 | -5.87405 | -0.32528 |
| H | 5.781624 | -7.01242 | -0.34242 |
| H | 0.955339 | -6.81698 | -0.42302 |
| H | 3.37959 | -6.9148 | -0.3829 |
| H | -1.45005 | -6.69565 | -0.46301 |
| H | -3.85582 | -6.60006 | -0.50228 |
| H | -6.27293 | -6.47922 | -0.54227 |
| C | 0.712669 | 1.460275 | 3.903843 |
| N | 0.546431 | 0.047595 | 3.560046 |
| C | -0.59251 | -0.59608 | 4.249702 |
| C | -0.54988 | -0.51855 | 5.753038 |
| C | -1.68043 | -0.27808 | 6.533945 |
| C | -1.57259 | -0.32787 | 7.923849 |
| C | -0.33086 | -0.61187 | 8.486791 |
| N | 0.772646 | -0.83978 | 7.759325 |
| C | 0.649166 | -0.79416 | 6.430057 |
| C | -1.86055 | -0.09434 | 3.523551 |
| C | -1.38033 | 0.02633 | 2.056515 |
| C | 0.171691 | -0.13525 | 2.148347 |
| H | 1.567069 | 1.857545 | 3.346927 |
| H | 0.931534 | 1.553381 | 4.970683 |
| H | -0.16342 | 2.094801 | 3.680524 |
| H | -0.49262 | -1.65462 | 3.994788 |
| H | -2.64001 | -0.08217 | 6.066215 |
| H | -2.43967 | -0.16825 | 8.55731 |
| H | -0.21415 | -0.6745 | 9.566853 |
| H | 1.548048 | -0.99308 | 5.850463 |
| H | -2.70313 | -0.78033 | 3.638261 |
| H | -2.17302 | 0.878094 | 3.91994 |
| H | -1.66278 | 0.990276 | 1.623054 |
| H | -1.81562 | -0.75316 | 1.424285 |
| H | 0.45757 | -1.14981 | 1.847184 |
| H | 0.72044 | 0.567211 | 1.51533 |

(Ⅵ)

| C | -1.40124 | -5.62693 | -0.48139 |
| --- | --- | --- | --- |
| C | -0.16518 | -4.98869 | -0.47352 |
| C | 1.005086 | -5.7483 | -0.44142 |
| C | -2.57398 | -4.87939 | -0.5131 |
| C | -0.10247 | -3.59164 | -0.49761 |
| C | -1.27615 | -2.84029 | -0.52931 |
| C | -2.51172 | -3.48117 | -0.53705 |
| C | -1.22365 | -1.44568 | -0.55338 |
| C | -0.02044 | -0.80475 | -0.54667 |
| C | 1.177882 | -1.54482 | -0.51468 |
| C | 1.129112 | -2.94381 | -0.49004 |
| C | 5.828329 | -5.94359 | -0.36032 |
| C | 7.063545 | -5.29951 | -0.35103 |
| C | 4.65507 | -5.19562 | -0.39292 |
| C | 7.124979 | -3.9068 | -0.37525 |
| C | 5.951098 | -3.1569 | -0.40866 |
| C | 4.716185 | -3.79929 | -0.4172 |
| C | 6.012108 | -1.76385 | -0.4334 |
| C | 7.264387 | -1.12522 | -0.42476 |
| C | 3.422798 | -5.84583 | -0.40122 |
| C | 2.245117 | -5.09779 | -0.43351 |
| C | 2.30618 | -3.69417 | -0.45788 |
| C | 3.544417 | -3.04479 | -0.44988 |
| C | 4.821191 | -1.01354 | -0.46664 |
| C | 3.61073 | -1.64233 | -0.47451 |
| C | 2.422326 | -0.89064 | -0.5072 |
| C | -6.22052 | -5.41067 | -0.56078 |
| C | -4.98225 | -4.77298 | -0.55271 |
| C | -3.80863 | -5.53126 | -0.52073 |
| C | -7.40024 | -4.64926 | -0.59323 |
| C | -4.92067 | -3.37667 | -0.57662 |
| C | -6.09834 | -2.63351 | -0.6085 |
| C | -7.34047 | -3.28935 | -0.61668 |
| C | -6.02755 | -1.23452 | -0.63208 |
| C | -4.81772 | -0.61316 | -0.62379 |
| C | -3.62778 | -1.36295 | -0.59187 |
| C | -3.68449 | -2.72375 | -0.56878 |
| H | -8.35077 | -5.14042 | -0.59959 |
| H | -8.24434 | -2.71734 | -0.64145 |
| C | -2.42494 | -0.71667 | -0.58451 |
| C | 0.069061 | 0.665618 | -0.57055 |
| C | -1.07695 | 1.399219 | -0.6018 |
| C | -1.00796 | 2.78834 | -0.62574 |
| C | 0.228104 | 3.426574 | -0.61787 |
| C | 1.398369 | 2.666969 | -0.58576 |
| C | -2.1807 | 3.535876 | -0.65745 |
| C | 0.29081 | 4.823629 | -0.64196 |
| C | -0.88287 | 5.57498 | -0.67366 |
| C | -2.11843 | 4.9341 | -0.6814 |
| C | 1.522395 | 5.471459 | -0.63438 |
| C | 7.3499 | 0.332388 | -0.44888 |
| C | 6.1591 | 1.078543 | -0.48137 |
| C | 6.221612 | 2.47168 | -0.50467 |
| C | 7.456829 | 3.115761 | -0.49538 |
| C | 5.048353 | 3.219648 | -0.53727 |
| C | 7.518262 | 4.508466 | -0.5196 |
| C | 6.344381 | 5.258372 | -0.55301 |
| C | 5.109468 | 4.615976 | -0.56155 |
| C | 4.906031 | 0.436257 | -0.49066 |
| C | 3.816081 | 2.569438 | -0.54557 |
| C | 2.6384 | 3.31748 | -0.57786 |
| C | 2.699465 | 4.721097 | -0.60223 |
| C | 3.937702 | 5.370481 | -0.59423 |
| C | 3.757604 | 1.171353 | -0.52167 |
| C | 1.325664 | 1.306663 | -0.56242 |
| C | 2.504533 | 0.537706 | -0.53 |
| C | -4.7291 | 0.880102 | -0.64964 |
| C | -5.87963 | 1.604763 | -0.6807 |
| C | -5.82723 | 3.004597 | -0.70513 |
| C | -4.58897 | 3.642281 | -0.69706 |
| C | -3.41534 | 2.884009 | -0.66508 |
| C | -3.47808 | 1.523244 | -0.64163 |
| C | -7.00696 | 3.76601 | -0.73758 |
| C | -4.52738 | 5.038599 | -0.72097 |
| C | -5.70506 | 5.781756 | -0.75285 |
| C | -6.94718 | 5.125912 | -0.76103 |
| C | -3.29121 | 5.691518 | -0.71313 |
| H | -7.95749 | 3.27485 | -0.74394 |
| H | -7.85105 | 5.697927 | -0.7858 |
| C | -2.33589 | 0.775118 | -0.61027 |
| C | -7.08234 | 0.969728 | -0.68811 |
| C | -7.16975 | -0.49683 | -0.66346 |
| H | -7.98244 | 1.547761 | -0.7124 |
| H | -8.12357 | -0.98169 | -0.67004 |
| H | -5.66358 | 6.850797 | -0.7711 |
| H | -3.24419 | 6.760331 | -0.73125 |
| H | -0.83401 | 6.643706 | -0.69203 |
| H | 1.567163 | 6.540358 | -0.65313 |
| H | 3.985567 | 6.439245 | -0.61306 |
| H | 6.392118 | 6.327137 | -0.57209 |
| H | 8.467704 | 5.001832 | -0.51252 |
| H | 8.359127 | 2.541221 | -0.46963 |
| H | 8.300769 | 0.822998 | -0.44194 |
| H | 8.159875 | -1.71038 | -0.40038 |
| H | 8.07442 | -3.41343 | -0.36817 |
| H | 7.965843 | -5.87405 | -0.32528 |
| H | 5.781624 | -7.01242 | -0.34242 |
| H | 0.955339 | -6.81698 | -0.42302 |
| H | 3.37959 | -6.9148 | -0.3829 |
| H | -1.45005 | -6.69565 | -0.46301 |
| H | -3.85582 | -6.60006 | -0.50228 |
| H | -6.27293 | -6.47922 | -0.54227 |
| C | 0.531267 | -1.69339 | 6.225529 |
| N | 0.471057 | -0.30904 | 6.696124 |
| C | -0.61683 | 0.477087 | 6.075124 |
| C | -0.58049 | 0.532518 | 4.570646 |
| C | -1.72604 | 0.448173 | 3.779201 |
| C | -1.61514 | 0.61497 | 2.398685 |
| C | -0.35581 | 0.855494 | 1.854897 |
| N | 0.761811 | 0.934193 | 2.592179 |
| C | 0.635613 | 0.778169 | 3.912789 |
| C | -1.9186 | 0.007548 | 6.76206 |
| C | -1.44836 | -0.28031 | 8.208908 |
| C | 0.111378 | -0.22693 | 8.121131 |
| H | 1.353765 | -2.20168 | 6.738478 |
| H | 0.742295 | -1.7059 | 5.153136 |
| H | -0.38969 | -2.27858 | 6.397002 |
| H | -0.43812 | 1.497951 | 6.423491 |
| H | -2.69745 | 0.282749 | 4.234115 |
| H | -2.49188 | 0.578036 | 1.759514 |
| H | -0.23503 | 1.006433 | 0.784053 |
| H | 1.546987 | 0.856629 | 4.501625 |
| H | -2.70763 | 0.761814 | 6.715396 |
| H | -2.3029 | -0.89924 | 6.282024 |
| H | -1.80187 | -1.25575 | 8.555893 |
| H | -1.82407 | 0.469165 | 8.9117 |
| H | 0.472287 | 0.732265 | 8.510303 |
| H | 0.606311 | -1.02246 | 8.684562 |

MD

| 0.903784 | 0.933186 | 0.170584 |
| --- | --- | --- |
| 0.036235 | -0.00238 | 0.164439 |
| 0.903176 | 0.131753 | 0.166858 |
| 0.035995 | 0.197003 | 0.162937 |
| 0.902359 | 0.329826 | 0.161757 |
| 0.035855 | 0.396754 | 0.162251 |
| 0.90213 | 0.529303 | 0.160237 |
| 0.035873 | 0.596846 | 0.162322 |
| 0.903128 | 0.731647 | 0.166484 |
| 0.03616 | 0.797561 | 0.16406 |
| 0.102474 | 0.930042 | 0.162163 |
| 0.235167 | -0.00479 | 0.15794 |
| 0.102263 | 0.129414 | 0.160725 |
| 0.23475 | 0.194092 | 0.155192 |
| 0.102395 | 0.329753 | 0.161604 |
| 0.235602 | 0.396056 | 0.16037 |
| 0.102594 | 0.530331 | 0.16308 |
| 0.23636 | 0.597976 | 0.165511 |
| 0.102587 | 0.730373 | 0.163036 |
| 0.236074 | 0.797368 | 0.1636 |
| 0.302209 | 0.929552 | 0.160773 |
| 0.43574 | -0.00343 | 0.161461 |
| 0.301093 | 0.126731 | 0.153557 |
| 0.434355 | 0.1931 | 0.152442 |
| 0.301449 | 0.327584 | 0.155918 |
| 0.434706 | 0.394027 | 0.154895 |
| 0.302766 | 0.530748 | 0.164338 |
| 0.43642 | 0.598322 | 0.16645 |
| 0.303057 | 0.731583 | 0.166379 |
| 0.436864 | 0.799388 | 0.169123 |
| 0.503168 | 0.931923 | 0.167156 |
| 0.636885 | -0.00057 | 0.169105 |
| 0.50169 | 0.128149 | 0.15707 |
| 0.635336 | 0.195561 | 0.158852 |
| 0.500914 | 0.326234 | 0.152177 |
| 0.634592 | 0.393771 | 0.154285 |
| 0.502341 | 0.529891 | 0.161907 |
| 0.636217 | 0.597999 | 0.165584 |
| 0.503745 | 0.733394 | 0.171374 |
| 0.637606 | 0.801382 | 0.174605 |
| 0.704164 | 0.934404 | 0.173767 |
| 0.837271 | 0.000309 | 0.171538 |
| 0.70277 | 0.13093 | 0.164493 |
| 0.836203 | 0.197669 | 0.164587 |
| 0.701531 | 0.327801 | 0.156342 |
| 0.835313 | 0.395608 | 0.159159 |
| 0.701973 | 0.529045 | 0.159627 |
| 0.835818 | 0.596733 | 0.161882 |
| 0.703926 | 0.733854 | 0.172672 |
| 0.837212 | 0.800192 | 0.171302 |
| 0.67517 | 0.48027 | 0.741822 |
| 0.577527 | 0.554535 | 0.598931 |
| 0.445728 | 0.471715 | 0.629781 |
| 0.377747 | 0.535348 | 0.711861 |
| 0.253428 | 0.452858 | 0.72881 |
| 0.200583 | 0.308458 | 0.664809 |
| 0.382321 | 0.325208 | 0.569333 |
| 0.656912 | 0.714247 | 0.718953 |
| 0.794744 | 0.756401 | 0.693428 |
| 0.786693 | 0.613639 | 0.589533 |
| 0.662788 | 0.501238 | 0.594686 |
| 0.263515 | 0.244494 | 0.585987 |
| 0.735784 | 0.43566 | 0.723373 |
| 0.581793 | 0.40542 | 0.755078 |
| 0.718625 | 0.577986 | 0.86203 |
| 0.563444 | 0.543961 | 0.47066 |
| 0.421339 | 0.648747 | 0.762582 |
| 0.198739 | 0.500242 | 0.792203 |
| 0.10386 | 0.240779 | 0.677464 |
| 0.431292 | 0.270807 | 0.50345 |
| 0.624182 | 0.772796 | 0.689777 |
| 0.648281 | 0.732841 | 0.849787 |
| 0.861812 | 0.820249 | 0.814899 |
| 0.828412 | 0.819228 | 0.623567 |
| 0.790633 | 0.594952 | 0.458187 |
| 0.864962 | 0.610334 | 0.63672 |
